# A Peptide-Based Method for ^13^C Metabolic Flux Analysis in Microbial Communities

**DOI:** 10.1371/journal.pcbi.1003827

**Published:** 2014-09-04

**Authors:** Amit Ghosh, Jerome Nilmeier, Daniel Weaver, Paul D. Adams, Jay D. Keasling, Aindrila Mukhopadhyay, Christopher J. Petzold, Héctor García Martín

**Affiliations:** 1 Physical Biosciences Division, Lawrence Berkeley National Laboratory, Berkeley, California, United States of America; 2 Joint BioEnergy Institute, Emeryville, California, United States of America; 3 Department of Bioengineering, University of California, Berkeley, Berkeley, California, United States of America; 4 Department of Chemical Engineering, University of California, Berkeley, Berkeley, United States of America; Hellas, Greece

## Abstract

The study of intracellular metabolic fluxes and inter-species metabolite exchange for microbial communities is of crucial importance to understand and predict their behaviour. The most authoritative method of measuring intracellular fluxes, ^13^C Metabolic Flux Analysis (^13^C MFA), uses the labeling pattern obtained from metabolites (typically amino acids) during ^13^C labeling experiments to derive intracellular fluxes. However, these metabolite labeling patterns cannot easily be obtained for each of the members of the community. Here we propose a new type of ^13^C MFA that infers fluxes based on peptide labeling, instead of amino acid labeling. The advantage of this method resides in the fact that the peptide sequence can be used to identify the microbial species it originates from and, simultaneously, the peptide labeling can be used to infer intracellular metabolic fluxes. Peptide identity and labeling patterns can be obtained in a high-throughput manner from modern proteomics techniques. We show that, using this method, it is theoretically possible to recover intracellular metabolic fluxes in the same way as through the standard amino acid based ^13^C MFA, and quantify the amount of information lost as a consequence of using peptides instead of amino acids. We show that by using a relatively small number of peptides we can counter this information loss. We computationally tested this method with a well-characterized simple microbial community consisting of two species.

## Author Summary

Microbial communities underlie a variety of important biochemical processes ranging from underground cave formation to gold mining or the onset of obesity. Metabolic fluxes describe how carbon and energy flow through the microbial community and therefore provide insights that are rarely captured by other techniques, such as metatranscriptomics or metaproteomics. The most authoritative method to measure fluxes for pure cultures consists of feeding the cells a labeled carbon source and deriving the fluxes from the ensuing metabolite labeling pattern (typically amino acids). Since we cannot easily separate cells of metabolite for each species in a community, this approach is not generally applicable to microbial communities. Here we present a method to derive fluxes from the labeling of peptides, instead of amino acids. This approach has the advantage that peptides can be assigned to each species in a community in a high-throughput fashion through modern proteomic methods. We show that, by using this method, it is theoretically possible to recover the same amount of information as through the standard approach, if enough peptides are used. We computationally tested this method with a well-characterized simple microbial community consisting of two species.

## Introduction

Microbial communities have radically altered Earth's chemical composition and are largely responsible for the biogeochemical cycling of energy and carbon on its surface [1]. Their activities underpin a variety of important biochemical processes ranging from lignocellulose degradation in termite guts [2] to gigantic underground cave formation [3]. Furthermore, they form the basis of industrial applications as diverse as wastewater treatment [4] or extraction of gold from mineral ore [5], to name a few. These industrial applications demand reliable performances, a condition which is not always fulfilled. Phosphorus extraction for wastewater treatment, for example, is a widely used microbially-mediated process which often suffers from upsets of unknown origin [6].

While the recent advent of metagenomics [7], metatranscriptomics [8] and metaproteomics [9] has revolutionized our understanding of microbial communities, these techniques provide a knowledge that is descriptive in nature, rather than predictive. Questions such as: “which species will become dominant if pH is altered?”, or “how will the community's metabolic activity affect the acetate levels of its environment” are, as of today, not answerable from just the knowledge of the genomes, transcripts, proteins and metabolites present in a microbial community. Tackling these questions requires detailed knowledge of how carbon and energy flow inside the microbial community.

The flow of mass and energy in a microbial community is described by metabolic fluxes, which are defined as the rate at which molecules proceed through each reaction per unit time [10]. The knowledge of metabolic fluxes for all reactions in all organisms in a microbial community plus the exchange fluxes between organisms provides a map of how carbon and electrons flow through the community's metabolism to enable its function. Metabolic fluxes for pure cultures have been studied through a variety of techniques including Flux Balance Analysis (FBA) [11], ^13^C Metabolic Flux Analysis (^13^C MFA) [10], elementary flux mode analysis [12] and extreme pathway analysis [13]. The capability of measuring and predicting metabolic fluxes has provided not only a better understanding of the microbial phenotype, but also the means to bioengineer microbes for the production of desirable chemical products [14].

Out of the flux analysis techniques mentioned above, only FBA has been extended to deal with microbial communities. An early attempt to model the metabolism of the mixed community involved in the Enhanced Biological Phosphorous Removal (EBPR) process met limited success due to the lack of accurate genomic information [15]. More recently, FBA has been used to study the symbiotic relationship of a mutualistic co-culture comprising a sulfate reducer (*Desulfovibrio vulgaris*) and a methanogen (*Methanococcus maripaludis*) [16]. The two-species model predicted several features of the syntrophic co-culture growth, including the ratios of abundance of the two species and of formate and hydrogen as electron donors. Other attempts have used the Dynamic Multi-species Metabolic Modeling (DMMM) framework [17], based on dynamic flux balance analysis [18], to model a *Clostridium acetobutylicum* and *Clostridium cellulolyticum* co-culture involved in consolidated bioprocessing (CBP) of cellulosic biomass [19], and the competition of *Rhodoferax* and *Geobacter* species in anoxic subsurface environments [17]. This framework was used to successfully predict chemostat growth and byproduct secretion for the CBP system. For the *Rhodoferax* and *Geobacter* competitive system, it predicted the dominance of either *Rhodoferax* vs *Geobacter* species under different rates of consumed acetate flux, in concordance with field observations. Recently, a multi-level and multi-objective optimization framework has been used to describe the metabolic contribution of individual microbial members in a community and the trade-offs between individual and community fitness criteria [20]. This framework was used to elucidate the metabolite exchange in a cellobiose-consuming microbial community composed of three different species, and to assess the level of sub-optimal growth in a phototrophic microbial mat.

Whereas FBA is probably the most popular flux analysis method, ^13^C MFA offers significant advantages over FBA. FBA determines fluxes by constraining them through the reaction stoichiometry from a genome-scale model and measured extracellular fluxes. Since these constraints are typically not enough to fully determine fluxes, they are calculated by assuming that metabolism has evolved to maximize growth rate (typically, but see [21] for other alternatives). While these fluxes can be easily calculated as the solution to a linear programming (LP) problem, the general applicability of the optimization principle has been questioned [21]–[23]. ^13^C MFA, on the other hand fully constrains fluxes by using the results of ^13^C labeling experiments on top of stoichiometry and flux measurement constraints. These experiments consist of feeding the culture with a defined ^13^C labeled substrate, wait for the label to distribute through metabolism and then measure the resulting labeling pattern (or Mass Distribution Vector [24], MDV) of selected metabolites through mass spectrometry or nuclear magnetic resonance. Each of these sets of labeling patterns for selected metabolites corresponds to a flux profile and ^13^C MFA solves the corresponding nonlinear programming (NLP) problem to determine the fluxes compatible with the collected data [25], [26]. While the network of reactions typically considered for ^13^C MFA is not comprehensive and usually only includes central carbon metabolism, it is considered the gold standard for flux quantification [21]. Flux inference through ^13^C MFA is often used for metabolic engineering and has found applications in understanding the biological production of alcohols, amino acids, organic acids, and proteins [27]. Furthermore, it has been used for the phenotypic characterization of non-model organisms such as phototrophic bacteria and archaea [28] and the unveiling of novel pathways [29].

**Figure 1:**
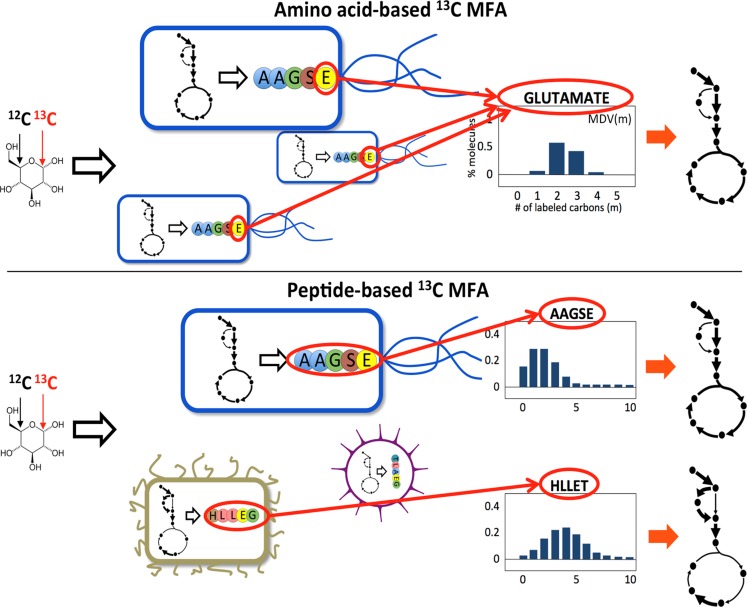
Overview of the traditional amino acid-based and the proposed peptide-based ^13^C MFA. For pure (top) and mixed cultures (bottom). A labeled carbon source is provided and the fluxes are derived from the ensuing amino acid labeling profiles. In the case of traditional ^13^C MFA for pure cultures, contributions from all cells, which are assumed to undergo similar metabolic activities, add up to produce the measured labeling profile (or MDV(*m*)  =  fraction of molecules with *m* ^13^C atoms incorporated). This measured labeling distribution is then used for the fit (see fig. 2). In the peptide-based method it is the peptide labeling distribution that is used for the fit (see fig. 2). Since peptides are composed of amino acids, its labeling distribution can be easily computed form the amino acid labeling profile, as shown in equation 13. The advantage of this approach is that the peptides can be separated and assigned to different species through standard proteomics techniques.

However, the standard ^13^C MFA procedure, based on inferring fluxes from proteogenic amino acid or intracellular metabolite labeling cannot distinguish contributions from different species in a microbial community (see Figure 1) and is hence challenging to perform for microbial communities. Previous attempts have targeted amino acids from reporter proteins giving ^13^C labeling patterns for subpopulation specific intracellular fluxes [30], [31], but this approach is not generalizable to all species in a microbial community. While it is, in principle, possible to separate species from a microbial community, obtain labeling patterns from each species and apply traditional ^13^C MFA, this is presently very time-consuming, cannot be done in a high-throughput manner and needs a different approach for each community. Single cell metabolomics coupled with cell sorting may be able to change this in the future [32]. In the meantime, we propose to circumvent this limitation by inferring fluxes from peptide labeling instead of amino acid labeling. This approach has the advantage that the peptides can be reliably attributed to different species (Figure 1) by using general high-throughput proteomic techniques which are applicable to any community for which sequence is available [33] [9], [34]. Moreover, in the same way that fluxes can be derived from amino acid labeling, we will show that they can be derived from the peptide labeling obtained from proteomic analysis [35], since peptides are composed of amino acids and their labeling determines that of the peptide (Figure 1).

In the next section, we will explain the traditional (amino acid-based) version of ^13^C MFA and compare it to the peptide-based version that we introduce here. By using peptide instead of amino acid labeling, fluxes are less constrained: i.e. more distinct flux profiles are compatible with the labeling data for peptides than amino acids. We will quantify this effect through an information content measure that will be explained after the new peptide-based method, and we will close the methods section explaining how we obtained the peptide sequences used to test the method. The results and discussion section will present a comparison between the amino acid and the peptide-based methods using data from the Keio collection multi-omics study [36], explore how the peptide-based method responds to noise in the peptide labeling, and investigate how information content is lost and recovered depending on the number of peptides used and their length. We will then apply the method to the simple microbial community mentioned above. We will finish by briefly discussing further challenges in making metafluxomics (i.e. the comprehensive study of metabolic fluxes in a microbial community) a reality.

## Methods

### Amino acid-based ^13^C Metabolic Flux Analysis (^13^C MFA)

^13^C MFA uses the result of ^13^C labeling experiments to determine intracellular metabolic fluxes for a variety of organisms. ^13^C labeling experiments consist of feeding a culture of the organism (*Escherichia coli* in this case) a labeled carbon source. The uptake and subsequent metabolizing of this carbon source confers internal metabolites a specific labeling pattern which depends highly on the intracellular metabolic fluxes. The solution to the inverse problem of finding which fluxes best fit the measured labeling patterns is called ^13^C MFA. Reviews and detailed explanations of the method can be found in previous publications [10], [25], [26]. The ^13^C MFA algorithm requires the following as inputs: a model of metabolism which includes carbon transition information (the fate of each carbon for each reaction [25]), measured values for extracellular fluxes (e.g, the uptake rate of glucose, and the excretion rate of metabolites), and the labeling pattern of each of the metabolites measured after the labeling experiment, typically through gas chromatography-mass spectrometry (GC-MS) [37], liquid chromatography-mass spectrometry (LC-MS) [38], or nuclear magnetic resonance (NMR) spectroscopy [39]. The labeling pattern is expressed in terms of the Mass Distribution Vector [24], [40] MDV(m), which is defined as the fraction of molecules with m = 0,1,2,3… labeled carbons. In this case, we used amino acid labeling measured through GC-MS and measured extracellular fluxes from the Keio collection multi-omics study for the *E. coli* rf05 strain [36], [40]. In order to calculate the labelling pattern corresponding to a flux profile, we used the EMU method [41]. We solved the inverse problem by using the CONOPT nonlinear solver in the GAMS (version 9.1) modeling environment to solve the optimization problem defined by the following equations as reproduced from reference [42]:
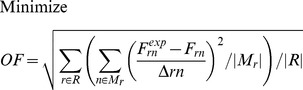
(1)

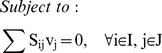
(2)




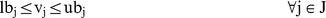
(3)

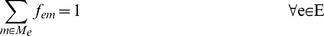
(4)

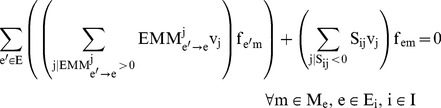
(5)

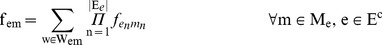
(6)

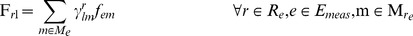
(7)


**Sets.**

I = {i}:Set of metabolites

J = {j}:Set of fluxes

E = {e}:Elementary Metabolite Units (EMUs)

E_c_ ⊂ E:Combined EMUs

E_i_ ⊂ E:EMUs from metabolite 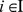


E_e_ ⊂ E:EMUs that produce combined EMU e

E_meas_ ⊂ E:EMUs corresponding to measured EMUs

R = {r}: Set of measured GC-MS fragments.

R_e_ ⊂ R:Measured GC-MS fragment related to EMU e

W_em_:Set of every possible mass isotopomer multiplet of E_e_ that produces the mass. isotopomer m of e

M_e_:m values for MDV of emu e: 0, 1,…, number of carbons in e

M_r_:m values for MDV of measured fragment r

**Parameters.** 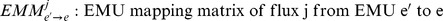



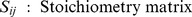
 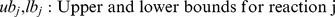



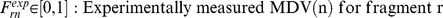



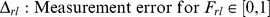



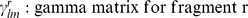


**Free variables.**


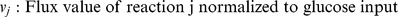



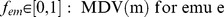



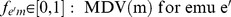



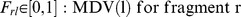


where *w* refers to the tuple:
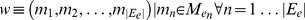


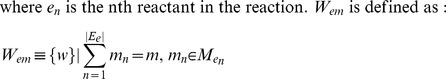
(8)


Equations 4 and 5 represent the normalization and the isotopomer balance respectively. Equation 6 represents the combination of Elementary Metabolite Units. Equation 7 adds the contributions of the non-carbon backbone atoms mass shift due to naturally occurring isotopic effects [43].

### Peptide based ^13^C Metabolic Flux Analysis

The amino acid-based ^13^C MFA method outlined above is challenging and not applicable to microbial communities. A sample obtained from a mixed community would contain a mixture of amino acids from all cells in the system and the labeling obtained through the usual analytical methods (GC-MS, LC-MS, NMR) would therefore be an average of all the labeling distributions corresponding to each cell (see Figure 1). For pure cultures this is not problematic since it is assumed that all cells undergo similar metabolic activity and fluxes obtained from this average amino acid labeling are a good representation of the metabolic fluxes from each cell. The fact that one can fit the metabolite labeling profiles using a single model [41], [44] (Toya *et al* 2010, Antoniewicz *et al* 2007) supports this assumption. In the case of a microbial community, we expect different species to display different metabolic activities: in fact, that is what makes microbial communities interesting. One might initially naively imagine that the fluxes obtained from the average amino acid labeling would provide the average flux profile if used in equations 1–8 above. This is not the case since the mapping of metabolic fluxes from amino acid labeling is highly nonlinear [45] and the average amino acid labeling does not correspond to the labeling of the average flux distribution. To see this, take 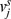 to be the flux for reaction j for species *s*, 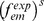 is a vector containing the labeling distribution for species *s*, and 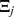 the non-linear function that maps the labeling distribution 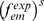 in through 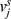 the solution of equations 1–8 above:
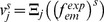
(9)


Then, the fluxes obtained by using the average amino acid labeling pattern would be:
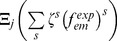
(10)


if we assume the fraction of species in the community to be 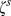.

The average flux profile would be:
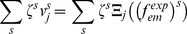
(11)


Notice that since omega is non-linear, these two are not necessarily the same:
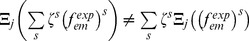
(12)


and, hence, the fluxes obtained from the average labeling distribution are not the same as the average flux. However, if a significant fraction of the bacterial species share metabolic activities, this approach may be feasible [46].

Unlike amino acids, peptides can be separated through modern proteomics methods and assigned to a species of a known genome, even in the case of labeled feeds [34], [35]. Since peptides are composed of amino acids, their MDVs can be obtained from the amino acid labeling through a convolution, or Cauchy product [47]. If we define 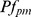 to be the experimentally measured MDV for isotopomer mass *m* for peptide *p*, and 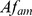 to be the MDV for amino acid *a∈ A = {A, C, D, E, F, G, H, I, K, L, M, N, P, Q, R, S, T, V, W, Y}* (amino acid set), this can be expressed as:
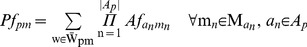
(13)


where 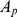 is the set of amino acids in peptide p, 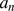
is the amino acid in position *n* in the peptide and 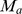
is the set of *m* values for the MDV for amino acid *a*. The tuple *w* is defined as:
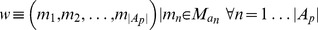


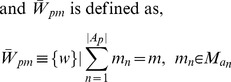
(14)


However, since the representation of equation 13 in the GAMS modeling system is not computationally efficient, we opted for a breakdown of the convolution into:
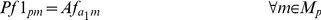
(15)

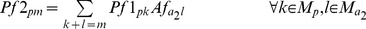
(16)




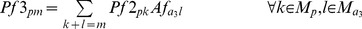
(17)



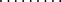




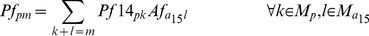
(18)


The variable 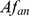 is obtained from the amino acid carbon backbone MDV by adding the contributions of the non-carbon backbone atoms mass shift due to naturally occurring isotopic effects [43]:
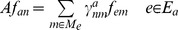
(19)


where 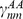 is matrix *A* from equation 19 in Wahl *et al* [43] for amino acid AA and 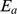 is the EMU that corresponds to amino acid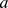. For peptides smaller than 15 amino acids, empty spaces in the input peptides sequences are filled with “dummy” amino acid represented by X. For these dummy amino acids, an identity matrix was used as 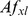. For example, peptide labeling for peptide number one, with five amino acids (1: ‘VLAYRXXXXXXXXXX’) can be derived as:



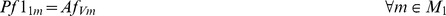


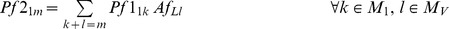


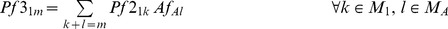


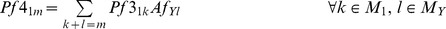


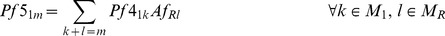


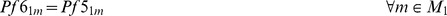


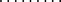


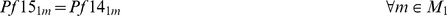



**Figure 2:**
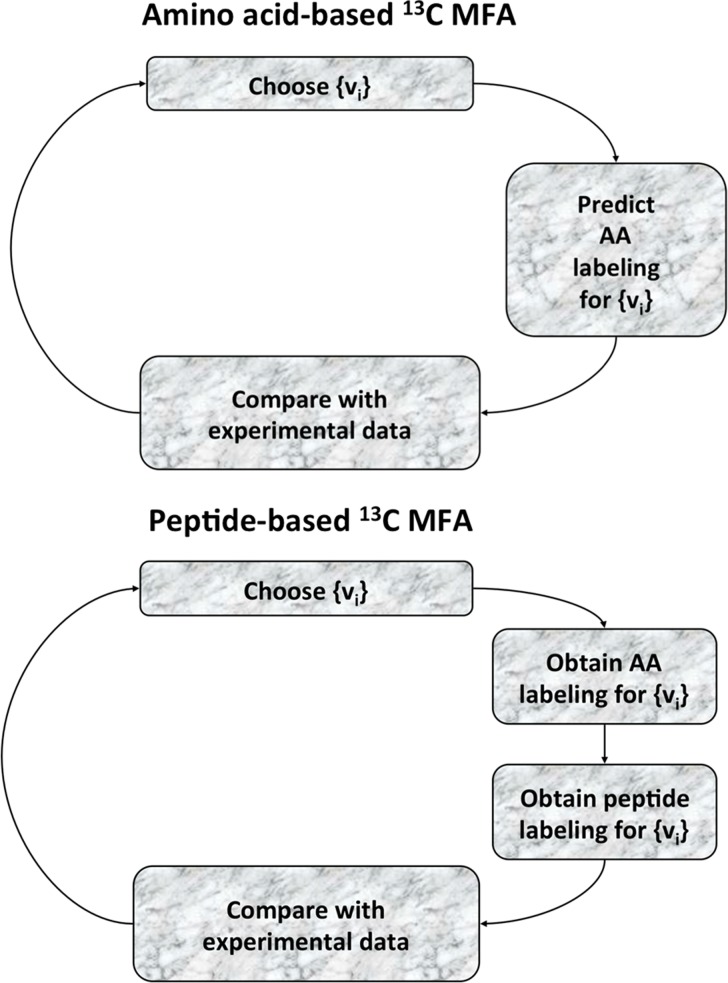
Schematic representation of the algorithmic differences between the amino acid-based and the peptide-based methods. The amino acid-based and the peptide-based methods are expressed in equations 1–8 and 20–31 respectively, and solved through GAMS. For pure cultures (top), a set of initial fluxes {v_i_} is chosen and the expected amino acid labeling is calculated. This computationally generated labeling is compared with the experimentally obtained labeling and the difference is quantified as an objective function to be minimized: OF({v_i_}). A new set of fluxes is then chosen so as to decrease the error function. The procedure is continued recursively until the calculated labeling is within the experimental error of the experimental data. For the peptide-based method (bottom), the only difference is that the experimental information used for the fit is the peptide labeling instead of the amino acid labeling. The peptide labeling is obtained from the amino acid labeling through equation 13.



These constraints are added to the ^13^C MFA constraints above in order to generate the expected labeling pattern for specific peptides. By comparing the results with the experimentally measured peptide labeling pattern we can use the same approach as for ^13^C MFA to infer fluxes (Figure 2). The fluxes that best match the experimental peptide labeling are obtained by solving the following NLP optimization problem fluxes by using the CONOPT nonlinear solver in GAMS (version 9.1):

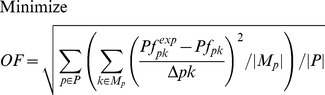
(20)

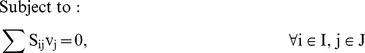
(21)

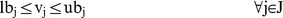
(22)




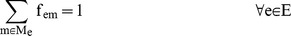
(23)

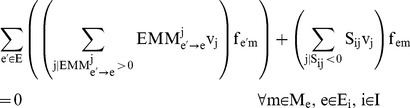
(24)

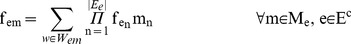
(25)

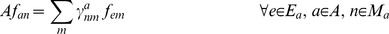
(26)

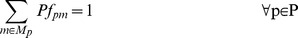
(27)

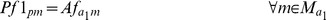
(28)




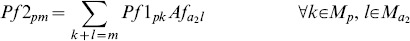
(29)

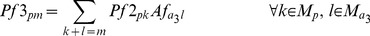
(30)

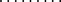


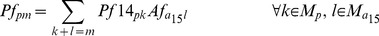
(31)


**Sets.**

I = {i}:Set of metabolites

J = {j}:Set of fluxes

E = {e}:Elementary Metabolite Units (EMUs)

E^c^ ⊂ E:Combined EMUs

E_i_ ⊂ E:EMUs from metabolite 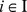


E_a_ ⊂ E:EMU corresponding to amino acid a

E_e_ ⊂ E:EMUs that produce combined EMU e

E_meas_ ⊂ E:EMUs corresponding to measured EMUs

W_em_:Set of every possible mass isotopomer multiplet of E_e_ that produces the mass. isotopomer m of e

M_e_:m values for MDV of emu e: 0, 1,…, number of carbons in e

M_p_:m values for MDV of peptide p

M_a_:m values for MDV of amino acid a

P = {p} Set of measured peptides

A = {A, C, D, E, F, G, H, I, K, L, M, N, P, Q, R, S, T, V, W, Y}, amino acids

**Parameters.**

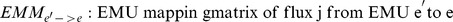


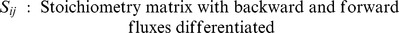


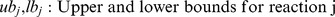


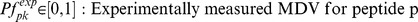


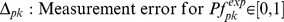


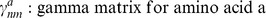



**Free variables.**


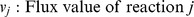



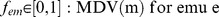



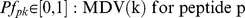



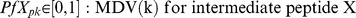



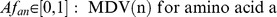


By using these extra constraints, we saw a three-fold increase in computational time compared to amino acid procedure. This computational time increases for longer peptides and for larger amounts of peptides, but remains within 10-fold of the initial time.

Our source code in GAMS is publicly available on figshare.

(http://dx.doi.org/10.6084/m9.figshare.1119727).

### Information content

**Figure 3:**
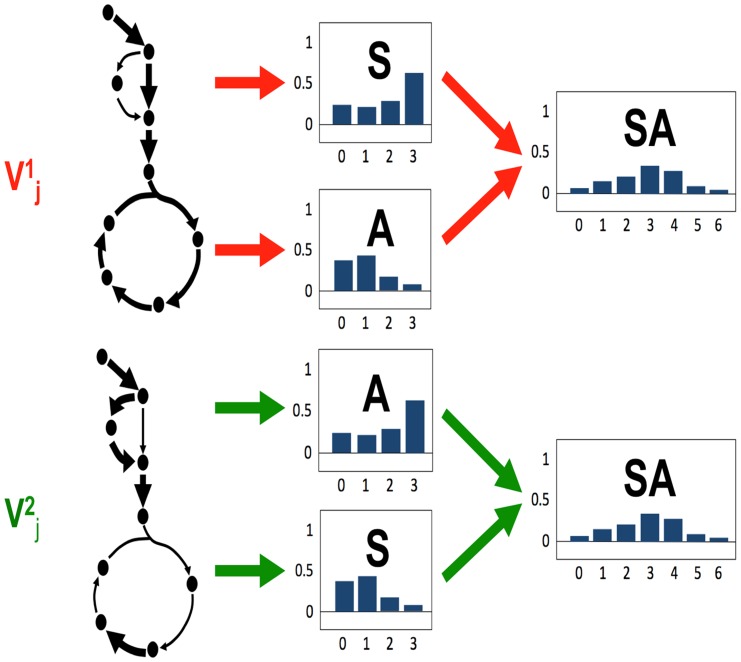
Graphical representation of information loss. A loss of information may be expected when using peptide-based ^13^C MFA relative to amino acid-based ^13^C MFA. Imagine two different flux profiles 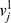 and 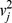 which generate different amino acid MDVs for Serine (S) and Alanine (A). One can recover 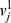 and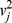 from the different amino acid labeling of S and A. However, in this example case, the MDV for the peptide obtained by combining both amino acids is the same, making it impossible to tell the flux profiles apart from the peptide labeling alone. In general, the convolution in equation 13 loses track of which amino acid the labeling patterns come from. As we can see in Figure 6 this loss of information can be countered by using more peptides.

The main disadvantage of using peptide over amino acid labeling is the loss of information: as can be observed in Figure 3, it is possible that different flux profiles producing different amino acid labeling profiles give rise to the same peptide labeling profile. Hence, one might think that the peptide-based method is less discerning than the amino acid-based one. We will show later that this is not the case, but in order to prove that conclusively we need to define the concept of flux information content (FIC).

**Figure 4:**
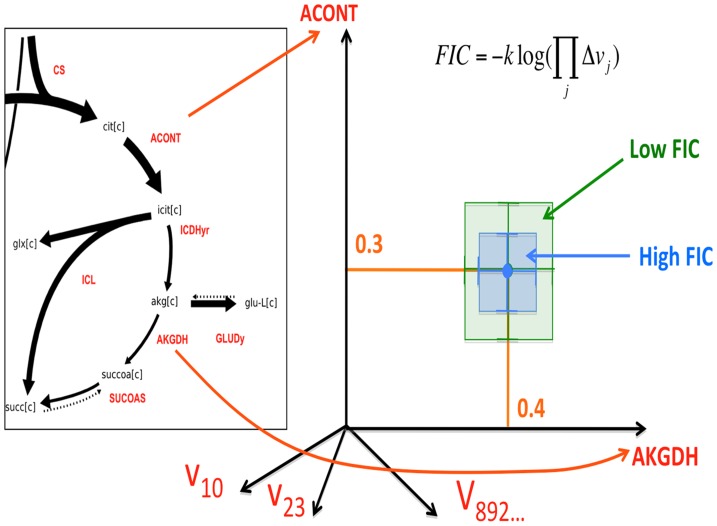
The Flux Information Content (FIC) for an experimental data set (amino acid-based or peptide-based): Represents how constrained fluxes are by this data set, and is inversely proportional to the logarithm of the volume of the phase space compatible with the experimental data set. The flux phase space or solution space is an imaginary space in which each coordinate axis corresponds to a different reaction. Each point in the phase space corresponds to a flux profile: the value of the flux is the coordinate in the corresponding coordinate axis (0.3 for ACONT and 0.4 for AKGDH in this example). The volume of the phase space compatible with experimental data is approximated by the volume of the hypercube given by the allowable ranges for each reaction. The allowable flux range for each flux for each reaction is obtained by finding the maximum and minimum values compatible with the experimental data set. A large allowable phase space corresponds to a higher indetermination of the flux profile and, hence, a low FIC. Conversely, a higher value of the FIC indicates that the flux is more effectively constrained by the experimental data.

The goal in defining the FIC is to try to quantify the degree of “constraint” that our experimental data introduces in the obtained flux profiles: i.e. how many flux profiles are compatible with the gathered experimental data? In order to do so we will use the concept of flux phase space (or solution space [48]). Imagine a coordinate space where each axis represents the value of a flux in your reaction network, with each point representing a flux profile as depicted in Figure 4. We will quantify the fraction of phase space 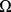 which is compatible with the current constraints involving measured fluxes, stoichiometry and labeling patterns from either amino acids or peptides.

The FIC will therefore be defined mirroring entropy [49] as:
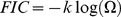
(32)


where k is a constant of inconsequential value for our purposes. Hence, the more space compatible with the current constraints indicate less constraining power by the current experimental data and less information on the state of the systems (lower FIC) while, conversely, a higher value of FIC indicates less volume Ω compatible with the experimental constraints and more information on the state of the system.

The precise value of Ω as defined above is difficult to calculate [50], so we will make an approximation that is still valid for our purposes:
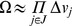
(33)


where the phase volume has been approximated by the enveloping box (see Figure 4), and
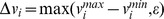



where ε = 0.01 is a lower bound and 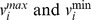 are the maximum and minimum values of flux allowed while constraining the computationally determined labeling value to be within the error bounds of the experimentally determined data:
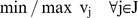
(34)

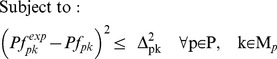
(35)

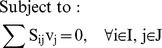
(36)

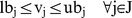
(37)




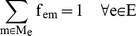
(38)

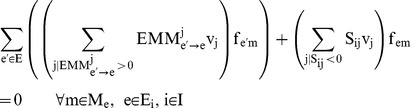
(39)

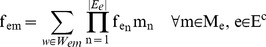
(40)

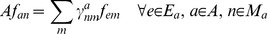
(41)

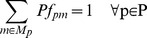
(42)




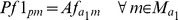
(43)




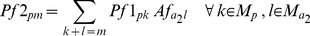
(44)

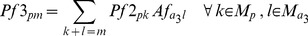
(45)

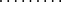


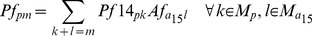
(46)


**Sets.**

I = {i}:Set of metabolites

J = {j}:Set of fluxes

E = {e}:Elementary Metabolite Units (EMUs)

E^c^ ⊂ E:Combined EMUs

E_i_ ⊂ E:EMUs from metabolite 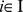


E_a_ ⊂ E:EMU corresponding to amino acid a

E_e_ ⊂ E:EMUs that produce combined EMU e

E_meas_ ⊂ E:EMUs corresponding to measured EMUs

W_em_:Set of every possible mass isotopomer multiplet of E_e_ that produces the mass. isotopomer m of e

M_e_:m values for MDV of emu e: 0, 1,…, number of carbons in e

M_p_:m values for MDV of peptide p

M_a_:m values for MDV of amino acid a

P = {p} Set of measured peptides

A = {A, C, D, E, F, G, H, I, K, L, M, N, P, Q, R, S, T, V, W, Y}, amino acids

**Parameters.**

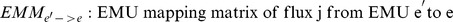




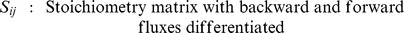


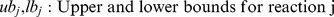


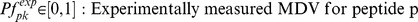


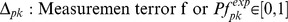


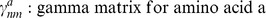



**Free variables.**

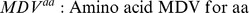


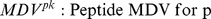


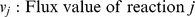


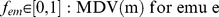


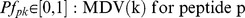


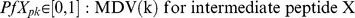


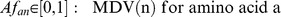



Hence, the FIC then becomes:
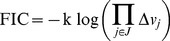
(47)


### Generation of peptide labeling test data

In order to compare the amino acid and peptide-based methods we used the data from the Keio collection multi-omics study [36]. We use the measured extracellular flux data and the labeling information for amino acids Ala, Asp, Gly, Glu, Ile, Met, Pro, Phe, Ser, Val and Tyr from *E. coli* rf05 strain to calculate fluxes for the reactions shown in Table S1 in this paper through ^13^C MFA by solving the optimization problem given in equations 1–8. Since we included biosynthesis reactions for all twenty amino acids, we obtained labeling information for all of them. Although the labeling information for the amino acids not mentioned above is not available in the Keio data set, the remaining amino acids share the same precursors as those mentioned above, so their MDVs could be easily derived. From the amino acid labeling we obtained the target peptide labeling by using equation 13. This data set was used as target peptide labeling distribution 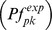 in equation 20.

The peptide sequence (see Table 1) was obtained from the *E. coli* genome [51] by simulating trypsin digestion using Protein Prospector [52]. Sets of peptides formed by 5, 10 and 15 amino acids were chosen such that they were not only unique, but had different amino acid composition. This is important because peptide labeling depends only on composition, not on amino acid order. A fourth group was chosen as a mix of the peptides of 5, 10 and 15 amino acids. Fluxes were obtained by solving the optimization problem defined in equation 20–31.

**Table 1:** The Peptide sequences from trypsin digestion of *E. coli* proteins.

5AA	10AA	15AA	Mixed group
GWQAK	EFVESLETPR	DVIYHIETYDVTTIR	GWQAK
LGLQK	RTADHVISAR	VNPVVPEVVNQVCFK	LGLQK
VAASK	SILANVEQIK	SIYVAYTGGTIGMQR	VAASK
DIFTK	TIADFTTNDR	LHYLLSQELDTETIR	DIFTK
VLAYR	ELIVASSYSK	WVASQITGEVTLELR	VLAYR
LPNPR	NPEAMAASLK	HEMSEFMIACGFDYK	LPNPR
QEVDR	AIEVVGGAAK	AVEAAGDVDVLLLDK	QEVDR
YQLLK	ADGVIFQTAV	QLALFEPTLVVQALK	YQLLK
FGAGK	QADAAVIAAK	LSAVVNLLNQALGDR	FGAGK
SDASK	IWLDADLLNK	ALVGSGIEAQVNGER	SDASK
LAQVK	AVASACAANK	NDDVLGVIALQDTLR	LAQVK
KLLTK	FTESGEGTGR	VLTSLVSWVVSFIPR	KLLTK
GEMER	AAALAAADAR	GEFVSIFDCDHVPTR	GEMER
QFLDK	AVGQLGLMCR	VYLNDELMGVLPVTK	QFLDK
NTSVK	TTVTSGGLQR	DSEALGALGQAYSQK	NTSVK
DETGK	FGHGSAQHVR	LELIDPNNPDVVAAR	DETGK
EQVLR	DLNIDPATLR	GLGGSSLINGMCYIR	EQVLR
IYAQK	CVEQLANWHK	IAADGQVNVALSGER	EFVESLETPR
VFALR	FDSVLNEAVK	LSGQTIEVTSEYLFR	RTADHVISAR
TFMVR	EGFHVVTPNK	VNWLGLGPQENYPDR	SILANVEQIK
IIEPR	MSVIAQAGAK	YYPNHEAVDFYGHYK	TIADFTTNDR
LLGIR	FGGSSLADVK	GISTSDLQPHGVMGK	ELIVASSYSK
DWAAK	NIGAFVVVTR	NASETGSIYSSMTLK	NPEAMAASLK
NFEGR	YPFLLSNGNR	GFLPFAPEADFWVGK	AIEVVGGAAK
YLQGR	RLGQDAAPEK	ALENELDGFTFEDNK	ADGVIFQTAV
QPWVK	SAASVAHWQK	QSYFHDFFNYAGIHR	QADAAVIAAK
YVFLK	VIASNGEDLE	KSATIAVVGPLADSK	IWLDADLLNK
LEMER	MMTTMLEVAK	TAEWAAEICGVGAAK	AVASACAANK
ACGVK	QAIQYLLDLR	VSWDEALDLIHQQHK	FTESGEGTGR
GLTTK	VVGGWNGESK	GTNASHVLVLIDGVR	AAALAAADAR
HVAER	WFDSQALMLR	NDVSDLIDYDDHTLK	AVGQLGLMCR
NFPNR	ADFLCGTGQK	DNDEDSPVYIATVPK	TTVTSGGLQR
AELAK	IDEIPFDFER	VGEWLVTPSINQISR	FGHGSAQHVR
DAISR	VPAGATSVDR	ADNGTITSGDAAMCK	DLNIDPATLR
LTQAR	EYTLSGSYTF	VSGGSDEMQILTLGR	DVIYHIETYDVTTIR
DLMDK	IGAIMVPINA	TLMVQPPSANDQQHR	VNPVVPEVVNQVCFK
DITLR	STGEVMGVGR	LMLPAWLGAGTALQK	SIYVAYTGGTIGMQR
MELGK	SVGEVMAIGR	YLGIGDYESWSEADK	LHYLLSQELDTETIR
DTLSR	GGVATIGVTR	LTIVPAQTSAEDVLK	WVASQITGEVTLELR
DLMEK	TLPNFPIEGR	SALLVLEDGTQFHGR	HEMSEFMIACGFDYK
RTGGK	ALSVPCSDSK	VAEVGITGLNADFLR	AVEAAGDVDVLLLDK
SIPLK	ATLEDLGQAK	TPASFEPSIDYVVTK	QLALFEPTLVVQALK
IGNEK	AWSASTVYVK	FSTVQGGAGSADTVR	LSAVVNLLNQALGDR
LLEEK	QNYSVSHNGR	DPSLSLYAIPDGDVK	ALVGSGIEAQVNGER
HISYK	AQAVGAADSL	FLELCNAGLSVEDIK	NDDVLGVIALQDTLR
GATER	AGGASSFLAD	EMTHAGELEHLTPER	VLTSLVSWVVSFIPR
QGPIR	QQETAVATMK	VVPEAFPEQSVPEGK	GEFVSIFDCDHVPTR
AQFIR	IPPESSNPLN	SIAQAMQHLSPQESK	VYLNDELMGVLPVTK
QANLR	EYQVVIDPQR	IWQQATAQAPALLDR	DSEALGALGQAYSQK
LEAIR	EGDVLCVIGR	VSLGNYFTGPGSIAR	LELIDPNNPDVVAAR

The peptide labeling profile of these sequences have been used to measure flux profiles for *E. coli* strain (rf05) using the peptide-based method.

The sequences in Table 1 are only an example of possible valid sequences. However, care must be taken that the sequences used are unique in each microbial community under study. Hence, the appropriate peptides sequences must be chosen for each species within a microbial community.

### Random noise representing experimental error in peptide measurement

The noisy target data used below was generated by adding a random value of amplitude Δ to the previously generated target data:
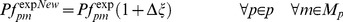
(48)


where 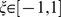 is random number drawn according to a constant probability distribution. Three set of noisy target data were generated with 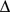 = 0.05, 0.08 and 0.10.

## Results/Discussion

### Comparison of intracellular fluxes obtained using the peptide and the amino acids based method for a pure culture

Our first goal is to compare results obtained through both methods discussed in this paper: the traditional amino acid-based ^13^C MFA and the peptide-based ^13^C MFA introduced here. We did this by using computationally-generated data based on the raw labeling data available for eleven amino acids from the Keio collection multi-omics study as an input. Since we included anabolic reactions for all twenty amino acids, we obtained labeling information for all of them. The remaining amino acids share the same precursors as those mentioned above and hence their MDVs could be easily derived. As explained in detail above, we compared fluxes obtained from amino acid-based ^13^C MFA with fluxes obtained from peptide-based ^13^C MFA where the target peptide labeling patterns had been derived from the amino acid-based ^13^C MFA. This approach allows us to compare the relative merits of each method starting from the same data.

**Figure 5:**
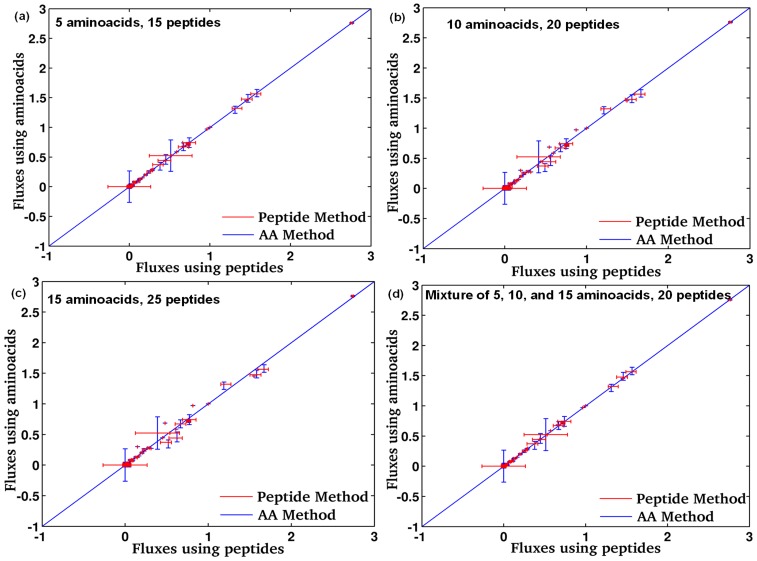
Comparison between flux profiles obtained through the amino acid-based and the peptide-based ^13^C FMA: 20 for 10 aa, 25 for 15 aa and 20 for the mixed group peptides. Peptide based ^13^C FMA flux profile obtained for the best fit for peptide lengths of (a) 5 amino acids (b) 10 amino acids (c) 15 amino acids and (d) mixture of 5, 10 and 15 amino acids. The information loss in longer peptides can be overcome adding more number of peptides to peptide-based ^13^C FMA.

Peptide-based ^13^C MFA produces the same results as amino acid-based ^13^C MFA, as can be seen in Figures 5 and S1, where 15 peptides each of them 5 amino acids long were used. Even the confidence intervals (Figure 5a and Table S2), which indicate how constrained fluxes are by the *E. coli* rf05 strain data, are similar. Two reactions (malic enzyme: ME1 and pyruvate kinase: PYK) show particularly large confidence intervals (∼0.5, normalized to glucose input).

The critical parameters to adjust in order to obtain similar results for peptide-based ^13^C MFA and amino acid-based ^13^C MFA are the number of peptides used, and their length. Hence, we also tested peptide-based ^13^C MFA with longer peptides: 10 and 15 amino acids long. Furthermore, since we expect that, in most practical cases, it would be hard to find peptides of the same exact length, we also considered a mixed group containing peptides consisting of 5, 10 and 15 amino acids. For a set peptide length, the more peptides used in the fit, the more accurate the flux determination is (Figure S2). Furthermore, for a set number of peptides, the longer the peptides are, the worse the results become in terms of recovering the intracellular fluxes (Figure S3). A detailed explanation of the reasons for these trends is discussed in the “Information loss” section. These trends are important because they interlace with the metaproteomics requirements. On one hand, longer peptides add difficulty to the task of recovering the flux profiles; on the other hand they ease the task of uniquely assigning the peptide to a sequenced genome: the longer a peptide is the higher the chances it is unique to a species.

Determining the appropriate number of peptides for each length is, then, a non-trivial task. In our case we determined the number of peptides for each peptide length in Figure 5 by the number required to recover the information value for the amino acid case: 20 for 10 aa, 25 for 15 aa and 20 for the mixed group (see the next section for details). While for the 5 aa group all fluxes are similar (they fall on the diagonal in Figure 5), for the 10 aa group fluxes for five reactions were found to differ (off the diagonal): 2-Oxoglutarate dehydrogenase (AKGDH), isocitrate dehydrogenase (ICDHy), glyceraldehyde-3-phosphate dehydrogenase (GAPD), phosphoglycerate kinase (PGK) and pyruvate dehydrogenase (PDH). However, in all these cases the range of the confidence interval either intercepts the diagonal or is very close, indicating that the values of the flux through either method are within the confidence interval of each other. Fluxes related to reactions MDH & PPCK, however, are off the diagonal and have a very small confidence interval that does not intersect the diagonal line. This indicates that the same solution was not recovered for both methods, a consequence of information loss, as detailed in the next section. For the 15 aa group, a flux not included in the previous group now clearly falls off the diagonal: MALS. The confidence intervals for this flux is also fairly small and do not intersect the diagonal. As can be observed the longer the peptides are, the more peptides are needed and the less accurate the method is, a consequence of the loss of information due to longer peptides. Interestingly, the mixed group (with 5, 10 and 15 aa long peptides) presents very similar results to the shortest peptides (5 aa), indicating that the inclusion of a few short peptides in a group of longer peptides can considerably improve the accuracy of the method.

The size of the confidence intervals indicate which fluxes the peptide-based method has greater trouble determining. Confidence intervals are largest for the following set of reactions from the TCA cycle: malic enzyme (ME1), succinate dehydrogenase (SUCDi), AKGDH, ICDHy & aconitase (ACONT) and glycolysis: pyruvate kinase (PYK), PDH, PGK & GAPD. The maximum confidence interval is observed for reactions ME1 and PYK and the value is around 0.5. Confidence intervals range from 0.12-0.16 for succinate dehydrogenase (SUCDi), AKGDH, ICDHy, ACONT, PDH, PGK and GAPD reactions. Since confidence intervals were the same for both the amino acid and the peptide-based methods, this indicates that information loss is not responsible for this phenomenon, but rather the position of the reactions in the network.

### Method assessment in the presence of noise

The data used in the previous section was a “perfect data” set in the sense that peptide labeling exactly matched the result of the convolution of amino acid labeling in equation 13 using amino acid labeling data obtained from the initial flux profiles (see Methods). However, in any realistic peptide labeling data we would expect noise due to either the instrument or experimental conditions. In this section we would like to explore the maximum amount of noise level we could allow in order to recover the same fluxes as through the amino acid method. In order to do so, we added a random noise to each peptide MDV with a relative amplitude of 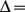
±0.05, ±0.08, and ±0.10 respectively (see Methods). Since the method for MDV measurement is the same for both amino acid and peptide analysis (i.e., mass spectrometry) we expect the measured noise in the amino acid and peptide labeling to be the same: ∼0.05.

As the amount of noise increase the fits to the peptide data deteriorate noticeably and the method cannot recover the results of the amino acid-based ^13^C MFA (Figures S4 and S5). However, the effect of noise is different depending on the amount of peptides used. For the case of 5 peptides and 10 amino acids per peptide the noise has a much more deleterious effect than for the case of 20 peptides and 10 amino acids per peptide (Figures S4 and S5). Confidence intervals for fluxes, however, become particularly large for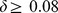, indicating that fluxes cannot be precisely estimated with particularly noisy data.

### Information loss

A possible drawback of the peptide-based method is the loss of information incurred by using peptides instead of amino acids when doing the data fit. After all, the convolution procedure loses track of where the different MDVs are coming from, and two peptides with the same composition and different MDVs for the amino acids may display the same peptide MDV (see Figure 3). We can quantify this effect through the definition of Flux Information Content (FIC, see method section) as an analog of entropy [49]. FIC quantifies how much the current experimental data constrains the flux profiles. A low FIC value indicates that the phase volume that is compatible with the experimental data is large, so the fluxes are rather unconstrained and the information value is low. Conversely, a high value of FIC indicates a small volume of allowable space and abundant information on the fluxes.

**Figure 6:**
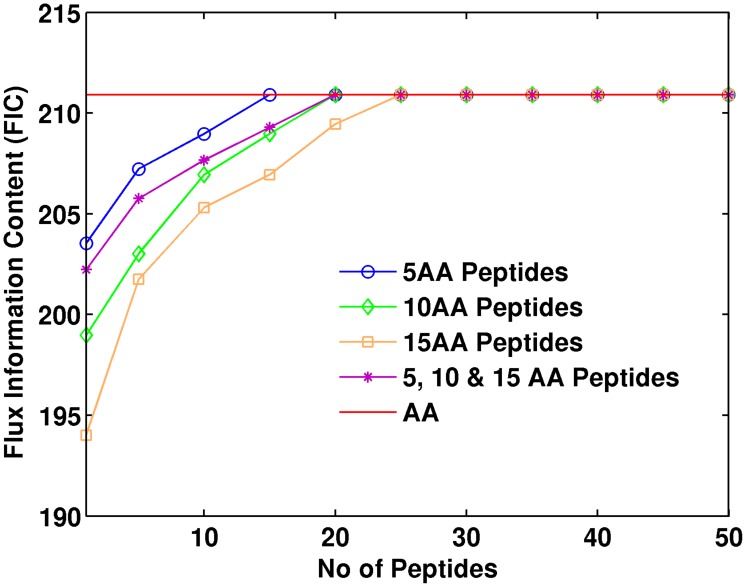
Flux Information Content (FIC) for the amino acid and peptide-based ^13^C MFA. The upper red line indicates the FIC for the amino acid-based method, the target. The different lines represent how the FIC grows as we add the labeling information of more peptides, therefore constraining fluxes more effectively. Each line corresponds to peptides of different length: 5, 10 and 15 amino acids long and, finally, a set of peptides of mixed lengths. As can be observed, the smaller the peptide the more FIC in the data set, with the mixed group being an interpolation between the 5 and 10 aa curves. This is not surprising in light of the intuitive argument for FIC loss given in Figure S2: the longer the peptides the more uncertainty there is about where the MDV is coming from. However, for all peptide lengths studied here there is a number of peptides that counter this information loss and makes peptide-based ^13^C MFA as informative as the amino acid-based method. This number of peptides is under 30 for all cases.

We calculated the FIC for flux profiles constrained by sets of different numbers of peptides and different peptide length (Figure 6). As expected, the FIC for 1 peptide is much lower than the FIC value obtained by constraining fluxes through the amino acid value. This is not surprising: the labeling pattern of one peptide contains much less information on fluxes than the labeling pattern of eight separate amino acids. As more peptides are added to the fit the FIC increases until the loss of information caused by using the peptide methods is completely countered and it reaches the FIC for the amino acid method. The number of peptides for which the peptide method FIC intercepts the amino acid FIC is different for different peptide lengths. As expected and discussed above, shorter peptides are more informative than longer peptides. For the peptide groups 5, 10 and 15 amino acids, as well as the mixed group, the number of peptides needed to match the amino acid FIC was 15, 20, 25 and 20 respectively. These represent a progression according with the peptide length, with the mixed group showing the same result as the group with the same average length (10 aa group). The FIC profile for the mixed group in Figure 6 corresponds to an interpolation of the 5 aa and the 10 aa groups profiles, a typical tryptic peptide length for most organisms. For example, the yeast proteome has an average tryptic peptide length of 8-9 amino acid residues and ∼97% of all tryptic peptides fall between 7–35 residues [53]. Since currently available shotgun or targeted proteomics methods can provide thousands of peptide identifications in a given sample by using multiple proteases [53], there is no obstacle to obtain the same FIC with the peptide method as the amino acid method. Hence, the information loss incurred by using peptides can be eliminated by using a large collection of peptides.

As an additional target flux profile we used the *pgi* knockout *E. coli* strain from the Keio collection multi-omics data. This flux profile is very different from the initial rf05 strain. We have used amino acid labeling data and measured extracellular fluxes for *pgi* knockout *E. coli* strain and then examined the ability of the peptide-based method to recover the flux distribution. We found that the method is still robust and the peptide requirement is 15 instead of 20 (Figure S6). The flux profile generated from peptide fitting has shown good correlation with amino acid based flux pattern for the *pgi* knockout *E. coli* strain (Figure S7).

We expect a trade-off between information loss for longer peptides and the ability to uniquely identify microbial species. As discussed above, the shorter the length of the peptide, the less flux information is lost. However, we expect that it will be more difficult to find unique short peptides the more diverse the community is. Hence, for the more complex microbial communities, we need to choose between a longer search for appropriate peptides for each species or using longer peptides with less capacity to resolve fluxes accurately.

### Peptide-based ^13^C Metabolic Flux Analysis of simple mutualistic microbial community

Next, we examined the capability of our peptide-based ^13^C MFA method to obtain the flux profile for a relatively simple and well-characterized syntrophic association between *D. vulgaris* and *M. maripaludis* [16], for which experimental data is available in the literature. The syntrophy-based mutualism between these two organisms is essential for the degradation of organic substrate. Lactate is used as a sole carbon source for *D. vulgaris* and produces CO_2_, formate and acetate, which serve as a growth substrate for *M. maripaludis*. Metabolic models and experimental growth data are available for the co-culture [16], [20], and have been used through FBA to produce feasible flux profiles for the individual species [16]. This FBA flux profile indicates that *D. vulgaris* growing optimally converts majority of the carbon present in the substrate lactate into acetate, formate and CO_2_. Acetate becomes a sole carbon source for *M. maripaludis* over the CO dehydrogenase pathway, consistent with published data and experimental results [16], [54]. Pyruvate-formate lyase (PFL) in *D. vulgaris* produces formate for utilization of *M. maripaludis*, but this formate does not contribute to its biomass growth and is just converted into methane.

We used the FBA flux profile and measured extracellular flux data for the microbial community to calculate the associated labeling information for amino acids. We obtained labeling information for all 20 amino acids for both organisms by including anabolic reactions for all of them (Table S3, S4), as we did for the *E. coli* model. From the amino acid labeling we obtained the target peptide labeling for both organisms in the same way as for the *E. coli* model. Peptide sequences (see Table S5) were obtained from the *D. vulgaris* and *M. maripaludis* genome by simulating trypsin digestion using Protein Prospector [52]. Sets of peptides formed by 10 amino acids were chosen such that they were unique in the community.

**Figure 7:**
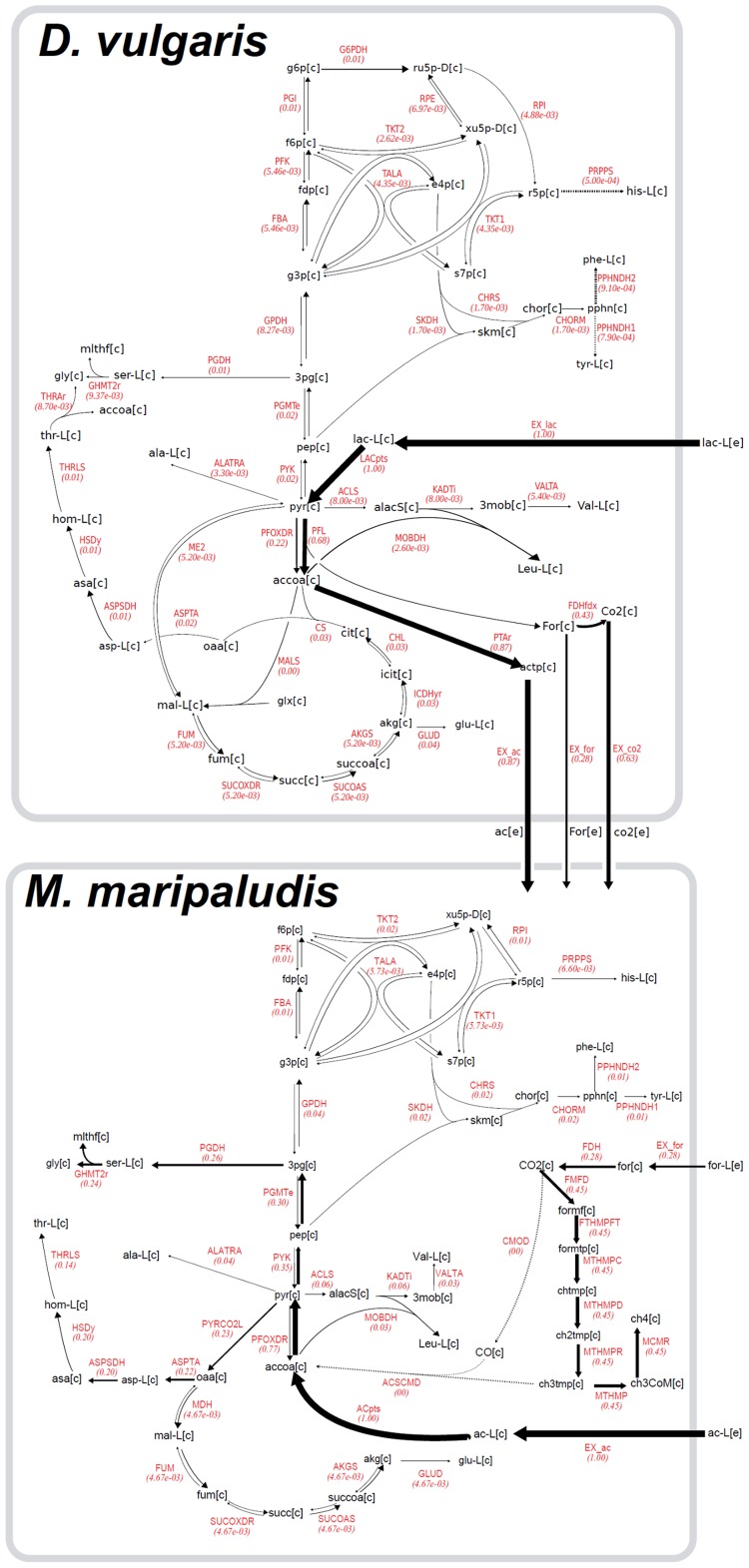
^13^C MFA Flux Map for *D. vulgaris* and *M. maripaludis* co-culture grown on lactate using peptide-based method for 10 amino acids and 20 peptides. The peptide-based method was able to recover the initial FBA flux profile used to derive peptide labeling, showing the feasibility of this method for a simple community. Lactate uptake flux was set as 1 µM/h; acetate, formate and CO_2_ were produced at a rate of 0.87 µM/h, 0.28 µM/h and 0.63 µM/h respectively. Best fit for flux is given on top red number for each reaction. Reversible reactions are indicated by double arrows.

We recovered the original FBA flux profile (Fig. 7) from the peptide-based ^13^C MFA by fitting the peptide labeling patterns obtained as described above. The particular structure of this flux profile for this specific community made the task easier: since there is no expected acetate, formate or CO_2_ flux coming back from *M. maripaludis* to *D. vulgaris*, both species may be compartimentalized and fluxes can be solved iteratively. In this fashion, the known lactate labeling, derived peptide labeling and known exchange fluxes for *D. vulgaris* were used to fit fluxes for this organism, according to equation 20–31. The acetate labeling obtained from solving this problem was used as input labeling, along with the derived peptide labeling to fit fluxes for *M. maripaludis*, according to equation 20–31 again. In this way, a “two-body” problem was solved as the combination of two “one-body” problems. The resulting fluxes were the same as the starting FBA solution. Furthermore, this solution produces a prediction of the acetate and peptide labeling that one should expect, were the FBA solution to apply to the real case. These predicted acetate and peptide labelings can be found in Figure S8.

### Further challenges for ^13^C-based metafluxomics

In this manuscript we have shown that we can use the labeling of peptides to derive intracellular metabolic fluxes as effectively as the amino acid labeling. In combination with the capability of assigning peptides to different species afforded by metaproteomics, this technique opens the door to determine fluxes for microbial communities. However, even in the simplest case of a homogenous environment, the method presented here is just the first step in making metafluxomics a reality, and a variety of hurdles need to overcome first.

^13^C MFA based on proteogenic amino acids (or peptides, for that matter) assumes a steady state both for fluxes and labeling patterns. If not met, the amino acid labeling pattern (and hence the peptide labeling) represents the labeling accumulation of all previous flux states, which cannot be deconvoluted using the method presented here. A possible solution to this would be to solve for all flux trajectories over time compatible with the current peptide labeling. The optimization-based method presented here, however, may not be applicable to this extended problem. Furthermore, even in the case of steady state for all community members the nonlinear optimization problem shown in equation 20–31 may be hindered by bad scaling properties for nonlinear problems. An alternative method to optimization, such as a Monte Carlo sampling [55] may provide a more scalable alternative.

Extracellular fluxes for each individual species in the community are difficult to obtain. However, they are not indispensable: measurement of total metabolites in the community will provide a constraint on all combined extracellular fluxes, although of a weaker nature than having measured fluxes for each species. Exchange of metabolites among the species in the community will have to be inferred through the labeling patterns by constraining flux exchanges compatible with the measured peptide labeling. This will require allowing multiple labeling sources in the model, a variation that is already available through the EMU method. A full determination of possible exchanges would require genome-scale models for all species in the community. The recently developed two-scale ^13^C MFA (2S-^13^C MFA, submitted) combines ^13^C labeling experiment data with genome-scale models and might be of use in this case.

Peptide labeling distributions for different members of a microbial community have already been obtained using labeled feeds [33]. Hence, we do not expect these measurements to be challenging to obtain. However, most algorithms used for peptide identification assume a natural abundance of the ^13^C isotope and are, therefore, not applicable to our cultures grown on ^13^C labeled feed. Nonetheless, recent developments in proteomics have surmounted the hurdle of peptide mass displacement and allow us to identify ^13^C labeled peptides accurately [9], [33], [34], [56]. The next step involves the development of an automated method to obtain the MDV as well as the peptide sequence.

### Conclusions and summary

The scope of potential applications of microbial communities in biotechnology is enormous. Microbial communities play key roles in the biological process on earth ranging from global carbon cycle to remineralisation of organic material and the breakdown of harmful substances. There is, therefore, a need to characterize the metabolic activities of such microbial ecosystems, and metabolic fluxes are measurable quantities of extreme importance towards that end. Here, we have introduced a computational method to obtain internal metabolic fluxes from the peptide labeling distribution following a ^13^C labeling experiment, instead of amino acid distributions. The advantage of this approach is that it is possible to assign peptides to each species in the microbial community using the peptide sequence and, simultaneously, infer metabolic fluxes using the peptide labeling.

We have, theoretically, shown that this method is equivalent to the standard amino acid-based ^13^C MFA method. But, in order for this to be the case, it is necessary to balance the length of the peptides used: too small and the metaproteomics method may fail to assign a species, too long and the fluxes may not be recovered. By using computational data, we have also set an upper bound to the level of noise allowed in the peptide distribution for the method to be effective. In order to compare the capacity of amino acid and peptide labeling information to constrain fluxes, we have defined the concept of FIC and demonstrated that one can eliminate the loss of information incurred by using peptide instead of amino acid labeling through the use of a set of peptides. The size of this set has been shown to be quite modest (15–25 peptides) compared to the available peptides in a metaproteomic study (thousands).

We have used peptide-based ^13^C MFA for a simple microbial community composed of two species. Amino acid labelling was inferred from the flux profile of the species present in a microbial system. We started with known flux distribution in a community of *D. vulgaris* and *M. maripaludis* and then calculated the peptide labeling *in silico*. From this peptide labeling it was possible to successfully recover inter-species metabolite transfer and the flux distribution for different species in a community.

However, there are still a variety of hurdles to be overcome before metafluxomics can become a reality that include the lack of a labeling steady state, the difficulty of measuring individual extracellular fluxes for each species and possible incomplete model reconstructions owing to the lack of full genomic coverage in metagenomic data.

## Supporting Information

Figure S1 ^13^C MFA Flux Map for the amino acid (top panel) and peptide-based (bottom panel) methods for 5 amino acids and 15 peptides. Best fit for flux is given on top red number for each reaction and confidence interval at the bottom. Cofactors and common metabolites are indicated by small arrows. Reversible reactions are indicated by double arrows. As can be observed, the results for both methods are virtually the same, validating the peptide-based approach.
(PDF)

Figure S2 Comparison between flux profiles obtained through amino acid and peptide-based ^13^C MFA for different peptide numbers. 10 amino acid long peptides were chosen for the fit and confidence interval has been estimated for (a) 1 peptide (b) 3 peptides (c) 5 peptides and (d) 15 peptides.
(PDF)

Figure S3 Comparison between flux profiles obtained through amino acid and peptide-based ^13^C MFA for different peptide lengths. 15 peptides were used for the fit and fluxes and confidence intervals were determined for the cases: (a) 5 amino acids (b) 10 amino acids (c) 15 amino acids and (d) mixture of 5, 10 and 15 amino acids.
(PDF)

Figure S4Peptide labeling data fit for *E. coli* rf05 strain for 20 peptides and 10 amino acids. Fits were obtained for experimentally measured peptide MDV's with following error rates (a) 0.05, (b) 0.08 and (c) 0.10. Red denotes the MDV for experimentally measured data, blue columns are the fit. Comparison between flux profiles measured through ^13^C MFA using amino acids and peptides, and fits were obtained for experimentally measured peptide MDV's with following error rates (d) 0.05, (e) 0.08 and (f) 0.10. The performance of the peptide-based method deteriorates strongly for 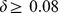.(PDF)

Figure S5 Peptide labeling data fit for the *E. coli* rf05 strain for 5 peptides and 10 amino acids. We have performed the same analysis as for figure S4 with following error rates (a) 0.05, (b) 0.08 and (c) 0.10, but using 5 peptides instead of 20. As can be observed the introduction noise has a much larger effect for this case than for the 20 peptides case.
(PDF)

Figure S6 Flux Information Content (FIC) for the amino acid and peptide-based ^13^C MFA for *pgi* knockout *E. coli* strain. The same trends as for the wild type can be observed, but in this case the number of required peptides is 15 instead of 20.
(PDF)

Figure S7 Comparison between flux profiles obtained through the amino acid-based and the peptide-based ^13^C FMA for *pgi* knockout *E. coli* stain. Peptide based ^13^C FMA flux profile obtained for the best fit for peptide lengths of 5 amino acids.
(PDF)

Figure S8 Peptide labeling labelling profile for peptide based 13C MFA obtained for *D. vulgaris* and *M. maripaludis* species in microbial community. Four 10 amino acids peptides sequences and MDV's were plotted for (a) DDFEPVNEVK, (b) NPEITDEENK, (c) GTALSGDDVR and (d) EGGTHLAGFK.
(PDF)

Table S1 The following list comprises the reactions included in peptide and amino acid based ^13^C MFA methods, along with their corresponding carbon transitions. Carbon transitions indicate the fate of each carbon in the reaction.
(PDF)

Table S2 Flux values for amino acids and peptide method for 5 amino acids and 15 peptides.
(PDF)

Table S3 List of *Desulfovibrio vulgar*is reactions included in peptide and amino acid based ^13^C MFA methods, along with their corresponding carbon transitions.
(PDF)

Table S4 List of *Methanococcus maripaludis* reactions included in peptide and amino acid based ^13^C MFA methods, along with their corresponding carbon transitions.
(PDF)

Table S5 The Peptide sequences from trypsin digestion of *D. vulgaris* and *M. maripaludis* proteins. The peptide labeling profile of these sequences have been used to measure flux profiles for *D. vulgaris* and *M. maripaludis* strains in a community using the peptide-based method.
(PDF)

## References

[1] Falkowski PG, Fenchel T, Delong EF (2008) The microbial engines that drive Earth's biogeochemical cycles. Science 320: 1034–1039 Available: http://www.ncbi.nlm.nih.gov/pubmed/18497287 Accessed 29 February 2012.. 18497287 10.1126/science.1153213

[2] Warnecke F, Luginbühl P, Ivanova N, Ghassemian M, Richardson TH, et al (2007) Metagenomic and functional analysis of hindgut microbiota of a wood-feeding higher termite. Nature 450: 560–565 Available: http://www.ncbi.nlm.nih.gov/pubmed/18033299 Accessed 6 March 2012.. 18033299 10.1038/nature06269

[3] Northup DE, Lavoie KH (2001) Geomicrobiology of Caves: A Review: 199–222.

[4] Oehmen A, Lemos P, Carvalho G, Yuan Z, Keller J, et al (2007) Advances in enhanced biological phosphorus removal: From micro to macro scale. Water Res 41: 2271–2300. 17434562 10.1016/j.watres.2007.02.030

[5] Rawlings DE (2002) Heavy metal mining using microbes. Annu Rev Microbiol 56: 65–91 10.1146/annurev.micro.56.012302.161052 12142493

[6] Gebremariam SY, Beutel MW, Christian D, Hess TF (2011) Research Advances and Challenges in the Microbiology of Enhanced Biological Phosphorus RemovalA Critical Review. Water Environ Res 83: 195–219 Available: http://www.ingentaconnect.com/content/wef/wer/2011/00000083/00000003/art00001. 21466069 10.2175/106143010x12780288628534

[7] Tringe SG, Rubin EM (2005) Metagenomics: DNA sequencing of environmental samples. Nat Rev Genet 6: 805–814. 16304596 10.1038/nrg1709

[8] Gilbert J a, Field D, Huang Y, Edwards R, Li W, et al (2008) Detection of large numbers of novel sequences in the metatranscriptomes of complex marine microbial communities. PLoS One 3: e3042 10.1371/journal.pone.0003042 18725995 PMC2518522

[9] Wilmes P, Bond PL (2006) Metaproteomics: studying functional gene expression in microbial ecosystems. Trends Microbiol 14: 92–97. 16406790 10.1016/j.tim.2005.12.006

[10] Tang YJ, Martin HG, Myers S, Rodriguez S, Baidoo EEK, et al (2009) Advances in analysis of microbial metabolic fluxes via (13)C isotopic labeling. Mass Spectrom Rev 28: 362–375 Available: http://eutils.ncbi.nlm.nih.gov/entrez/eutils/elink.fcgi?dbfrom=pubmed&id=19025966&retmode=ref&cmd=prlinks. 19025966 10.1002/mas.20191

[11] Varma A, Palsson BO (1994) Metabolic Flux Balancing: Basic Concepts, Scientific and Practical Use. Nat Biotech 12: 994–998 Available: 10.1038/nbt1094-994.

[12] Trinh C, Wlaschin A, Srienc F (2009) Elementary mode analysis: a useful metabolic pathway analysis tool for characterizing cellular metabolism. Appl Microbiol Biotechnol 81: 813–826 Available: 10.1007/s00253-008-1770-1. 19015845 PMC2909134

[13] Papin JA, Stelling J, Price ND, Klamt S, Schuster S, et al (2004) Comparison of network-based pathway analysis methods. Trends Biotechnol 22: 400–405 Available: http://www.sciencedirect.com/science/article/pii/S0167779904001751. 15283984 10.1016/j.tibtech.2004.06.010

[14] Yim H, Haselbeck R, Niu W, Pujol-Baxley C, Burgard A, et al (2011) Metabolic engineering of Escherichia coli for direct production of 1,4-butanediol. Nat Chem Biol 7: 445–452 Available: 10.1038/nchembio.580. 21602812

[15] Pramanik J, Trelstad PL, Schuler AJ, Jenkins D, Keasling JD (1999) Development and validation of a flux-based stoichiometric model for enhanced biological phosphorus removal metabolism. 33: 462–476.

[16] Stolyar S, Van Dien S, Hillesland KL, Pinel N, Lie TJ, et al (2007) Metabolic modeling of a mutualistic microbial community. Mol Syst Biol 3: 92 10.1038/msb4100131 17353934 PMC1847946

[17] Zhuang K, Izallalen M, Mouser P, Richter H, Risso C, et al (2011) Genome-scale dynamic modeling of the competition between Rhodoferax and Geobacter in anoxic subsurface environments. ISME J 5: 305–316 Available: 10.1038/ismej.2010.117. 20668487 PMC3105697

[18] Mahadevan R, Edwards JS, Doyle III FJ (2002) Dynamic Flux Balance Analysis of Diauxic Growth in Escherichia coli. Biophys J 83: 1331–1340 Available: http://www.sciencedirect.com/science/article/pii/S0006349502739039. 12202358 10.1016/S0006-3495(02)73903-9 PMC1302231

[19] Salimi F, Zhuang K, Mahadevan R (2010) Genome-scale metabolic modeling of a clostridial co-culture for consolidated bioprocessing. Biotechnol J 5: 726–738 Available: 10.1002/biot.201000159. 20665645

[20] Zomorrodi AR, Maranas CD (2012) OptCom: A Multi-Level Optimization Framework for the Metabolic Modeling and Analysis of Microbial Communities. PLoS Comput Biol 8: e1002363 10.1371/journal.pcbi.1002363 22319433 PMC3271020

[21] Schuetz R, Kuepfer L, Sauer U (2007) Systematic evaluation of objective functions for predicting intracellular fluxes in Escherichia coli. Mol Syst Biol 3. Available: 10.1038/msb4100162. PMC1949037 17625511

[22] Fischer E, Sauer U (2005) Large-scale in vivo flux analysis shows rigidity and suboptimal performance of Bacillus subtilis metabolism. Nat Genet 37: 636–640 Available: 10.1038/ng1555. 15880104

[23] Schuetz R, Zamboni N, Zampieri M, Heinemann M, Sauer U (2012) Multidimensional Optimality of Microbial Metabolism. Sci 336: 601–604 Available: http://www.sciencemag.org/content/336/6081/601.abstract. 10.1126/science.1216882 22556256

[24] Suthers PF, Burgard AP, Dasika MS, Nowroozi F, Van Dien S, et al (2007) Metabolic flux elucidation for large-scale models using 13C labeled isotopes. Metab Eng 9: 387–405 Available: http://www.sciencedirect.com/science/article/pii/S1096717607000316. 17632026 10.1016/j.ymben.2007.05.005 PMC2121621

[25] Wiechert W (2001) 13C Metabolic Flux Analysis. Metab Eng 3: 195–206 Available: http://www.sciencedirect.com/science/article/pii/S1096717601901879. 11461141 10.1006/mben.2001.0187

[26] Sauer U (2006) Metabolic networks in motion: 13C-based flux analysis. Mol Syst Biol 2 . Available: 10.1038/msb4100109. PMC1682028 17102807

[27] Toya Y, Shimizu H (n.d.) Flux analysis and metabolomics for systematic metabolic engineering of microorganisms. Biotechnol Adv. Available: http://www.sciencedirect.com/science/article/pii/S0734975013000839. 10.1016/j.biotechadv.2013.05.002 23680193

[28] Toya Y, Kono N, Arakawa K, Tomita M (2011) Metabolic Flux Analysis and Visualization†. J Proteome Res 10: 3313–3323 Available: 10.1021/pr2002885. 21815690

[29] Feng X, Tang K-H, Blankenship RE, Tang YJ (2010) Metabolic Flux Analysis of the Mixotrophic Metabolisms in the Green Sulfur Bacterium Chlorobaculum tepidum. J Biol Chem 285: 39544–39550 Available: http://www.jbc.org/content/285/50/39544.abstract. 20937805 10.1074/jbc.M110.162958 PMC2998096

[30] Rühl M, Hardt W-D, Sauer U (2011) Subpopulation-Specific Metabolic Pathway Usage in Mixed Cultures as Revealed by Reporter Protein-Based 13C Analysis. Appl Environ Microbiol 77: 1816–1821 Available: http://aem.asm.org/content/77/5/1816.abstract. 21216909 10.1128/AEM.02696-10 PMC3067264

[31] Shaikh AS, Tang YJ, Mukhopadhyay A, Keasling JD (2008) Isotopomer Distributions in Amino Acids from a Highly Expressed Protein as a Proxy for Those from Total Protein. Anal Chem 80: 886–890 Available: 10.1021/ac071445+. 18179240

[32] Rubakhin SS, Lanni EJ, Sweedler J V (2013) Progress toward single cell metabolomics. Curr Opin Biotechnol 24: 95–104 Available: http://www.sciencedirect.com/science/article/pii/S0958166912001760. 23246232 10.1016/j.copbio.2012.10.021 PMC3545069

[33] VerBerkmoes NC, Denef VJ, Hettich RL, Banfield JF (2009) Systems Biology: Functional analysis of natural microbial consortia using community proteomics. Nat Rev Micro 7: 196–205 Available: 10.1038/nrmicro2080. 19219053

[34] Huang X, Tolmachev A V, Shen Y, Liu M, Huang L, et al (2010) UNiquant, a Program for Quantitative Proteomics Analysis Using Stable Isotope Labeling. J Proteome Res 10: 1228–1237 Available: 10.1021/pr1010058. PMC3065106 21158445

[35] Pan C, Fischer CR, Hyatt D, Bowen BP, Hettich RL, et al. (2011) Quantitative Tracking of Isotope Flows in Proteomes of Microbial Communities. Mol Cell Proteomics 10 . Available: http://www.mcponline.org/content/10/4/M110.006049.abstract. 10.1074/mcp.M110.006049 PMC3069347 21285414

[36] Ishii N, Nakahigashi K, Baba T, Robert M, Soga T, et al (2007) Multiple High-Throughput Analyses Monitor the Response of E. coli to Perturbations. Sci 316: 593–597 Available: http://www.sciencemag.org/content/316/5824/593.abstract. 10.1126/science.1132067 17379776

[37] Christensen B, Nielsen J (1999) Isotopomer Analysis Using GC-MS. Metab Eng 1: 282–290 Available: http://www.sciencedirect.com/science/article/pii/S1096717699901179. 10937821 10.1006/mben.1999.0117

[38] Iwatani S, Van Dien S, Shimbo K, Kubota K, Kageyama N, et al (2007) Determination of metabolic flux changes during fed-batch cultivation from measurements of intracellular amino acids by LC-MS/MS. J Biotechnol 128: 93–111 Available: http://www.sciencedirect.com/science/article/pii/S0168165606007577. 17055605 10.1016/j.jbiotec.2006.09.004

[39] Malloy CR, Sherry AD, Jeffrey FM (1988) Evaluation of carbon flux and substrate selection through alternate pathways involving the citric acid cycle of the heart by 13C NMR spectroscopy. J Biol Chem 263: 6964–6971 Available: http://www.jbc.org/content/263/15/6964.abstract. 3284880

[40] http://ecoli.iab.keio.ac.jp/ (n.d.) No Title. Available: http://ecoli.iab.keio.ac.jp/.

[41] Antoniewicz MR, Kelleher JK, Stephanopoulos G (2007) Elementary metabolite units (EMU): A novel framework for modeling isotopic distributions. Metab Eng 9: 68–86 Available: http://www.sciencedirect.com/science/article/pii/S109671760600084X. 17088092 10.1016/j.ymben.2006.09.001 PMC1994654

[42] Suthers PF, Chang YJ, Maranas CD (2010) Improved computational performance of MFA using elementary metabolite units and flux coupling. Metab Eng 12: 123–128 Available: http://www.sciencedirect.com/science/article/pii/S1096717609000883. 19837183 10.1016/j.ymben.2009.10.002

[43] Wahl SA, Dauner M, Wiechert W (2004) New tools for mass isotopomer data evaluation in 13C flux analysis: Mass isotope correction, data consistency checking, and precursor relationships. Biotechnol Bioeng 85: 259–268 Available: 10.1002/bit.10909. 14748080

[44] Toya Y, Ishii N, Nakahigashi K, Hirasawa T, Soga T, et al (2010) 13C-metabolic flux analysis for batch culture of Escherichia coli and its pyk and pgi gene knockout mutants based on mass isotopomer distribution of intracellular metabolites. Biotechnol Prog 26: 975–992 Available: 10.1002/btpr.420. 20730757

[45] Wiechert W, Möllney M, Petersen S, de Graaf AA (2001) A Universal Framework for 13C Metabolic Flux Analysis. Metab Eng 3: 265–283 Available: http://www.sciencedirect.com/science/article/pii/S1096717601901880. 11461148 10.1006/mben.2001.0188

[46] Dijkstra P, Dalder JJ, Selmants PC, Hart SC, Koch GW, et al (2011) Modeling soil metabolic processes using isotopologue pairs of position-specific 13C-labeled glucose and pyruvate. Soil Biol Biochem 43: 1848–1857 Available: http://www.sciencedirect.com/science/article/pii/S0038071711001817.

[47] Arfken G (1985) Mathematical Methods for Physicists Academic Press. pp. 810–814.

[48] Wiback SJ, Mahadevan R, Palsson BØ (2004) Using metabolic flux data to further constrain the metabolic solution space and predict internal flux patterns: the Escherichia coli spectrum. Biotechnol Bioeng 86: 317–331 Available: 10.1002/bit.20011. 15083512

[49] Jaynes ET (1957) Information Theory and Statistical Mechanics. Phys Rev 106: 620–630 Available: http://link.aps.org/doi/10.1103/PhysRev.106.620.

[50] Wiback SJ, Famili I, Greenberg HJ, Palsson BØ (2004) Monte Carlo sampling can be used to determine the size and shape of the steady-state flux space. J Theor Biol 228: 437–447 Available: http://www.sciencedirect.com/science/article/pii/S0022519304000554. 15178193 10.1016/j.jtbi.2004.02.006

[51] Blattner FR, Plunkett G, Bloch CA, Perna NT, Burland V, et al (1997) The Complete Genome Sequence of Escherichia coli K-12. Sci 277: 1453–1462 Available: http://www.sciencemag.org/content/277/5331/1453.abstract. 10.1126/science.277.5331.1453 9278503

[52] Chalkley RJ, Baker PR, Huang L, Hansen KC, Allen NP, et al (2005) Comprehensive Analysis of a Multidimensional Liquid Chromatography Mass Spectrometry Dataset Acquired on a Quadrupole Selecting, Quadrupole Collision Cell, Time-of-flight Mass Spectrometer: II. New Developments in Protein Prospector Allow for Reliable and. Mol Cell Proteomics 4: 1194–1204 Available: http://www.mcponline.org/content/4/8/1194.abstract. 15937296 10.1074/mcp.D500002-MCP200

[53] Swaney DL, Wenger CD, Coon JJ (2010) Value of Using Multiple Proteases for Large-Scale Mass Spectrometry-Based Proteomics. J Proteome Res 9: 1323–1329 Available: 10.1021/pr900863u. 20113005 PMC2833215

[54] Shieh JS, Whitman WB (1987) Pathway of acetate assimilation in autotrophic and heterotrophic methanococci. J Bacteriol 169: 5327–5329 Available: http://jb.asm.org/content/169/11/5327.abstract. 3667534 10.1128/jb.169.11.5327-5329.1987 PMC213948

[55] Schellenberger J, Zielinski D, Choi W, Madireddi S, Portnoy V, et al (2012) Predicting outcomes of steady-state 13C isotope tracing experiments using Monte Carlo sampling. BMC Syst Biol 6: 9 Available: http://www.biomedcentral.com/1752-0509/6/9. 22289253 10.1186/1752-0509-6-9 PMC3323462

[56] Zimmer JSD, Monroe ME, Qian W-J, Smith RD (2006) Advances in proteomics data analysis and display using an accurate mass and time tag approach. Mass Spectrom Rev 25: 450–482 Available: 10.1002/mas.20071. 16429408 PMC1829209

