# Supplementary material for: A Peptide-Based Method for 13C Metabolic Flux Analysis in Microbial Communities
Source: PLoS Comput Biol. 2014 Sep 4;10(9):e1003827. doi: 10.1371/journal.pcbi.1003827 (PMC4154649; doi:10.1371/journal.pcbi.1003827)
Supplement: Figure S1 — 13C MFA Flux Map for the amino acid (top panel) and peptide-based (bottom panel) methods for 5 amino acids and 15 peptides. Best fit for flux is given on top red number for each reaction and confidence interval at the bottom. Cofactors and common metabolites are indicated by small arrows. Reversible reactions are indicated by double arrows. As can be observed, the results for both methods are virtually the same, validating the peptide-based approach. (PDF) [file pcbi.1003827.s001.pdf]

a) Flux Profile for AA Method

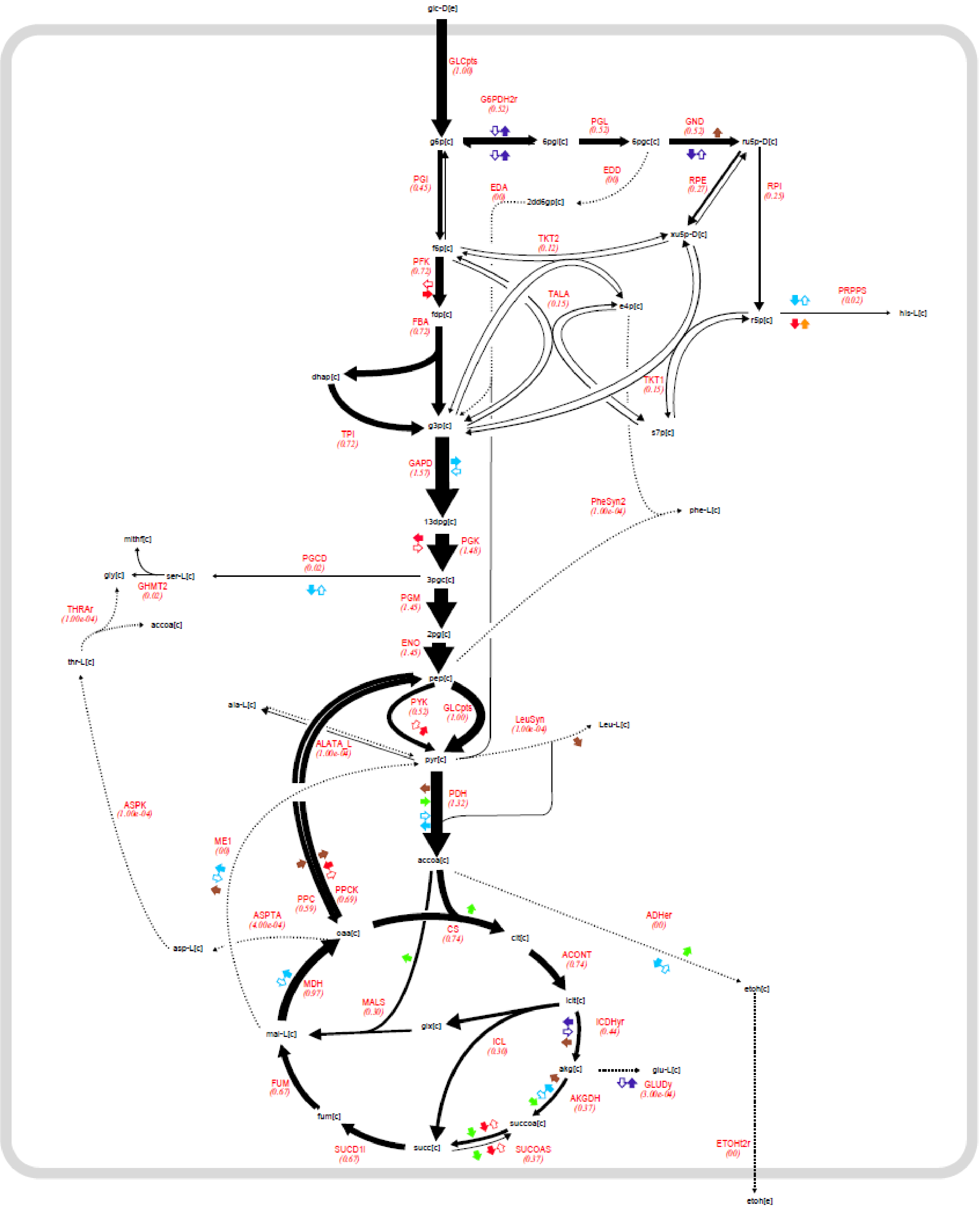

b) Flux Profile for Peptide Method

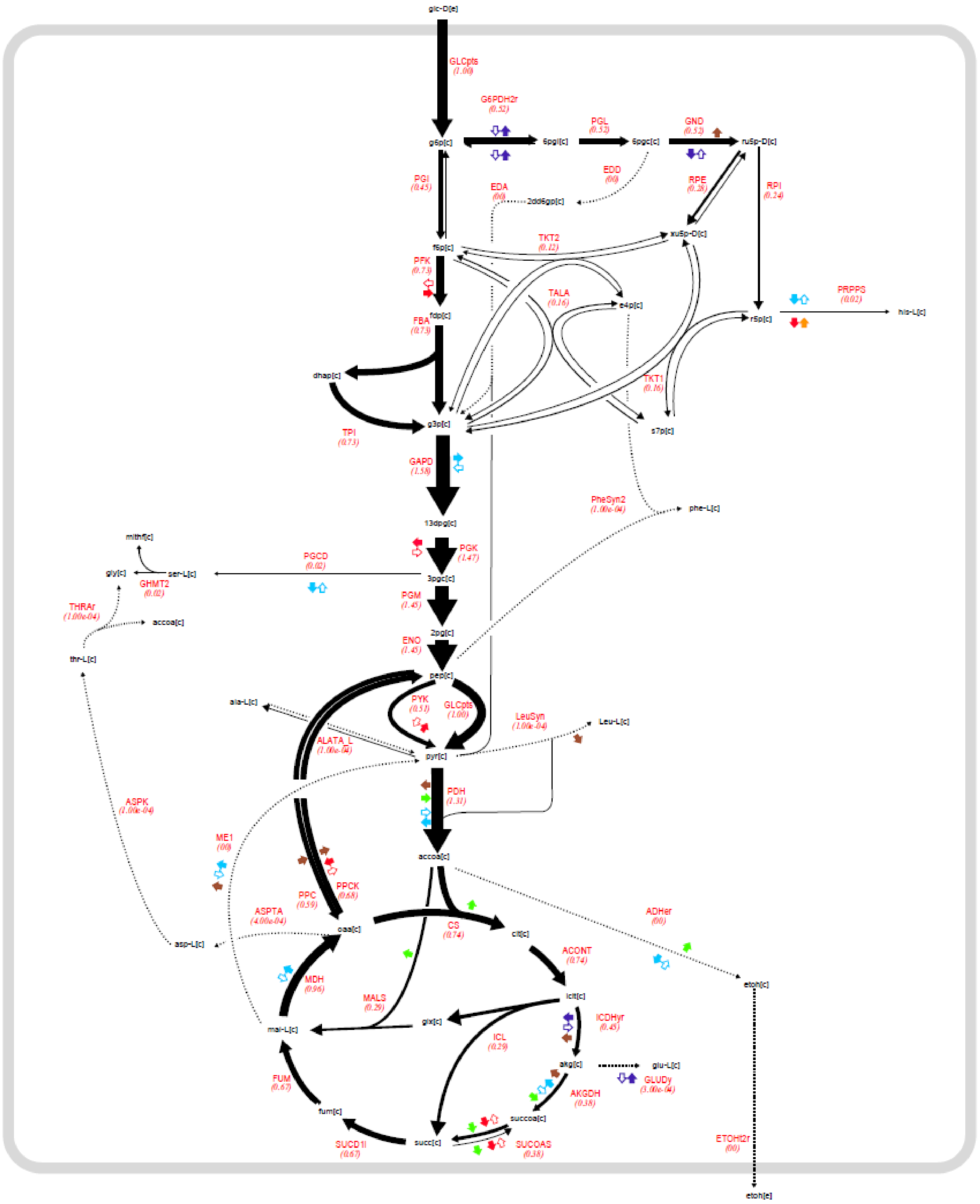

Figure S1. ^{13}C MFA Flux Map for the aminoacid (top panel) and peptide-based (bottom panel) methods for 5 aminoacids and 15 peptides. Best fit for flux is given on top red number for each reaction and confidence interval at the bottom. Cofactors and common metabolites are indicated by small arrows. Reversible reactions are indicated by double arrows. As can be observed, the results for both methods are virtually the same, validating the peptide-based approach.
