# Supplementary material for: A Peptide-Based Method for 13C Metabolic Flux Analysis in Microbial Communities
Source: PLoS Comput Biol. 2014 Sep 4;10(9):e1003827. doi: 10.1371/journal.pcbi.1003827 (PMC4154649; doi:10.1371/journal.pcbi.1003827)
Supplement: Figure S2 — Comparison between flux profiles obtained through amino acid and peptide-based 13C MFA for different peptide numbers. 10 amino acid long peptides were chosen for the fit and confidence interval has been estimated for (a) 1 peptide (b) 3 peptides (c) 5 peptides and (d) 15 peptides. (PDF) [file pcbi.1003827.s002.pdf]

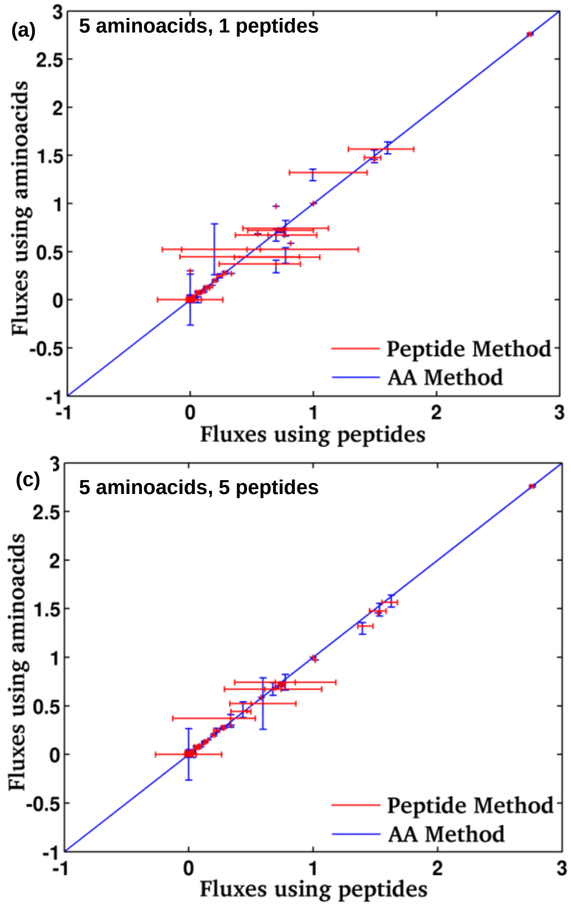

Figure S2. Comparison between flux profiles obtained through aminoacid and peptide-based ^{13}C MFA for different peptide numbers. 10 aminoacid long peptides were chosen for the fit and confidence interval has been estimated for (a) 1 peptide (b) 3 peptides (c) 5 peptides and (d) 15 peptides.
