# Supplementary material for: A Peptide-Based Method for 13C Metabolic Flux Analysis in Microbial Communities
Source: PLoS Comput Biol. 2014 Sep 4;10(9):e1003827. doi: 10.1371/journal.pcbi.1003827 (PMC4154649; doi:10.1371/journal.pcbi.1003827)
Supplement: Figure S3 — Comparison between flux profiles obtained through amino acid and peptide-based 13C MFA for different peptide lengths. 15 peptides were used for the fit and fluxes and confidence intervals were determined for the cases: (a) 5 amino acids (b) 10 amino acids (c) 15 amino acids and (d) mixture of 5, 10 and 15 amino acids. (PDF) [file pcbi.1003827.s003.pdf]

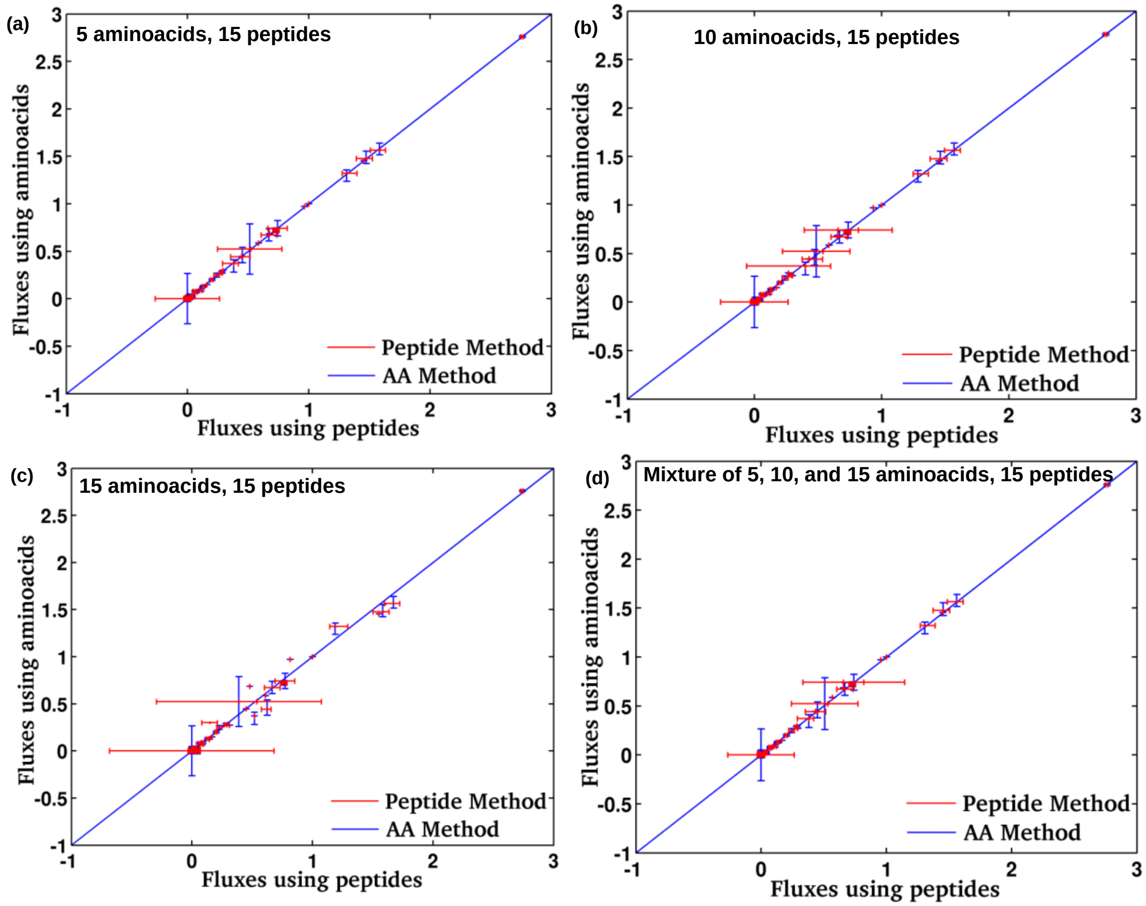

Figure S3. Comparison between flux profiles obtained through aminoacid and peptide-based ^{13}C MFA for different peptide lengths. 15 peptides were used for the fit and fluxes and confidence intervals were determined for the cases: (a) 5 aminoacids (b) 10 aminoacids (c) 15 aminoacids and (d) mixture of 5, 10 and 15 aminoacids.
