# Supplementary material for: A Peptide-Based Method for 13C Metabolic Flux Analysis in Microbial Communities
Source: PLoS Comput Biol. 2014 Sep 4;10(9):e1003827. doi: 10.1371/journal.pcbi.1003827 (PMC4154649; doi:10.1371/journal.pcbi.1003827)
Supplement: Figure S4 — Peptide labeling data fit for E. coli rf05 strain for 20 peptides and 10 amino acids. Fits were obtained for experimentally measured peptide MDV's with following error rates (a) 0.05, (b) 0.08 and (c) 0.10. Red denotes the MDV for experimentally measured data, blue columns are the fit. Comparison between flux profiles measured through 13C MFA using amino acids and peptides, and fits were obtained for experimentally measured peptide MDV's with following error rates (d) 0.05, (e) 0.08 and (f) 0.10. The performance of the peptide-based method deteriorates strongly for . (PDF) [file pcbi.1003827.s004.pdf]

(a)

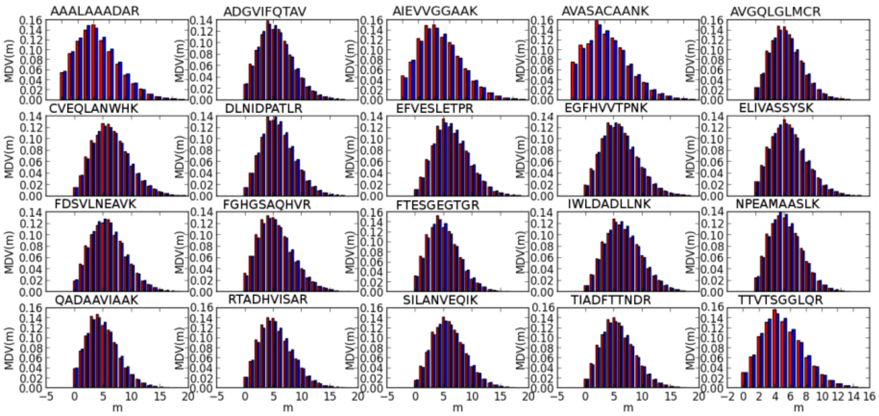
AAALAAADAR
ADGVIFQTAV
AIEVVGGAAK
AVASACAANK
AVGQLGLMCR
CVEQLANWHK
DLNIDPATLR
EFVESLETPR
EGFHVVTPNK
ELIVASSYSK
FDSVLNEAVK
FGHGSAQHVR
FTESGEGTGR
IWLDADLLNK
NPEAMAASLK
QADAAVIAAK
RTADHVISAR
SILANVEQIK
TIADFTTNDR
TTVTSGGLQR
MDV(m)
m

(b)

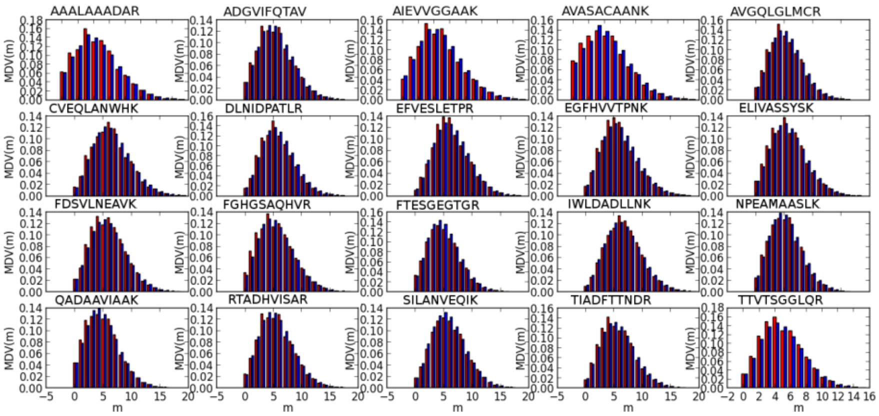
AAALAAADAR
ADGVIFQTAV
AIEVVGGAAK
AVASACAANK
AVGQLGLMCR
CVEQLANWHK
DLNIDPATLR
EFVESLETPR
EGFHVVTPNK
ELIVASSYSK
FDSVLNEAVK
FGHGSAQHVR
FTESGEGTGR
IWLDADLLNK
NPEAMAASLK
QADAAVIAAK
RTADHVISAR
SILANVEQIK
TIADFTTNDR
TTVTSGGLQR
MDV(m)
m

(c)

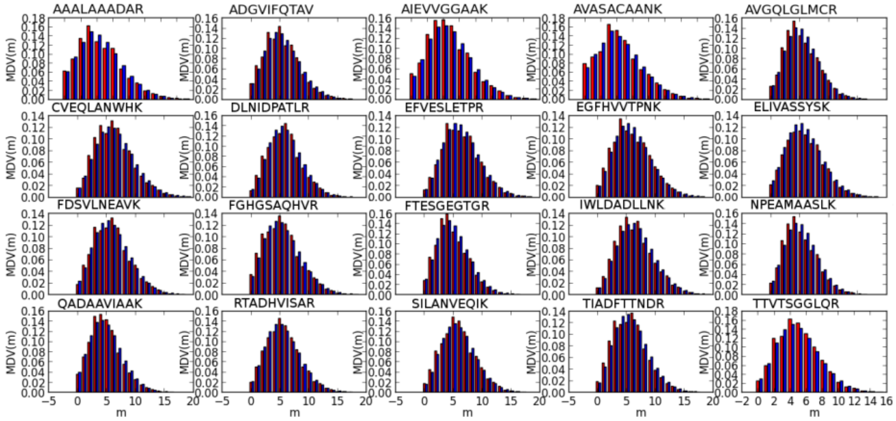
AAALAAADAR
ADGVIFQTAV
AIEVVGGAAK
AVASACAANK
AVGQLGLMCR
CVEQLANWHK
DLNIDPATLR
EFVESLETPR
EGFHVVTPNK
ELIVASSYSK
FDSVLNEAVK
FGHGSAQHVR
FTESGEGTGR
IWLDADLLNK
NPEAMAASLK
QADAAVIAAK
RTADHVISAR
SILANVEQIK
TIADFTTNDR
TTVTSGGLQR
MDV(m)
m

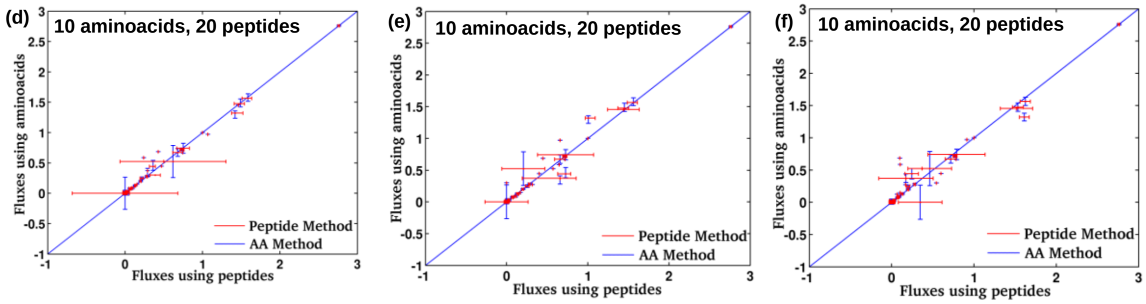

Figure S4. Peptide labeling data fit for *E. coli* rf05 strain for 20 peptides and 10 aminoacids. Fits were obtained for experimentally measured peptide MDV's with following error rates (a) 0.05, (b) 0.08 and (c) 0.10. Red denotes the MDV for experimentally measured data, blue columns are the fit. Comparison between flux profiles measured through ^{13}C MFA using aminoacids and peptides, and fits were obtained for experimentally measured peptide MDV's with following error rates (d) 0.05, (e) 0.08 and (f) 0.10. The performance of the peptide-based method deteriorates strongly for$\delta \geq 0.08$.
