# Supplementary material for: A Peptide-Based Method for 13C Metabolic Flux Analysis in Microbial Communities
Source: PLoS Comput Biol. 2014 Sep 4;10(9):e1003827. doi: 10.1371/journal.pcbi.1003827 (PMC4154649; doi:10.1371/journal.pcbi.1003827)
Supplement: Figure S5 — Peptide labeling data fit for the E. coli rf05 strain for 5 peptides and 10 amino acids. We have performed the same analysis as for figure S4 with following error rates (a) 0.05, (b) 0.08 and (c) 0.10, but using 5 peptides instead of 20. As can be observed the introduction noise has a much larger effect for this case than for the 20 peptides case. (PDF) [file pcbi.1003827.s005.pdf]

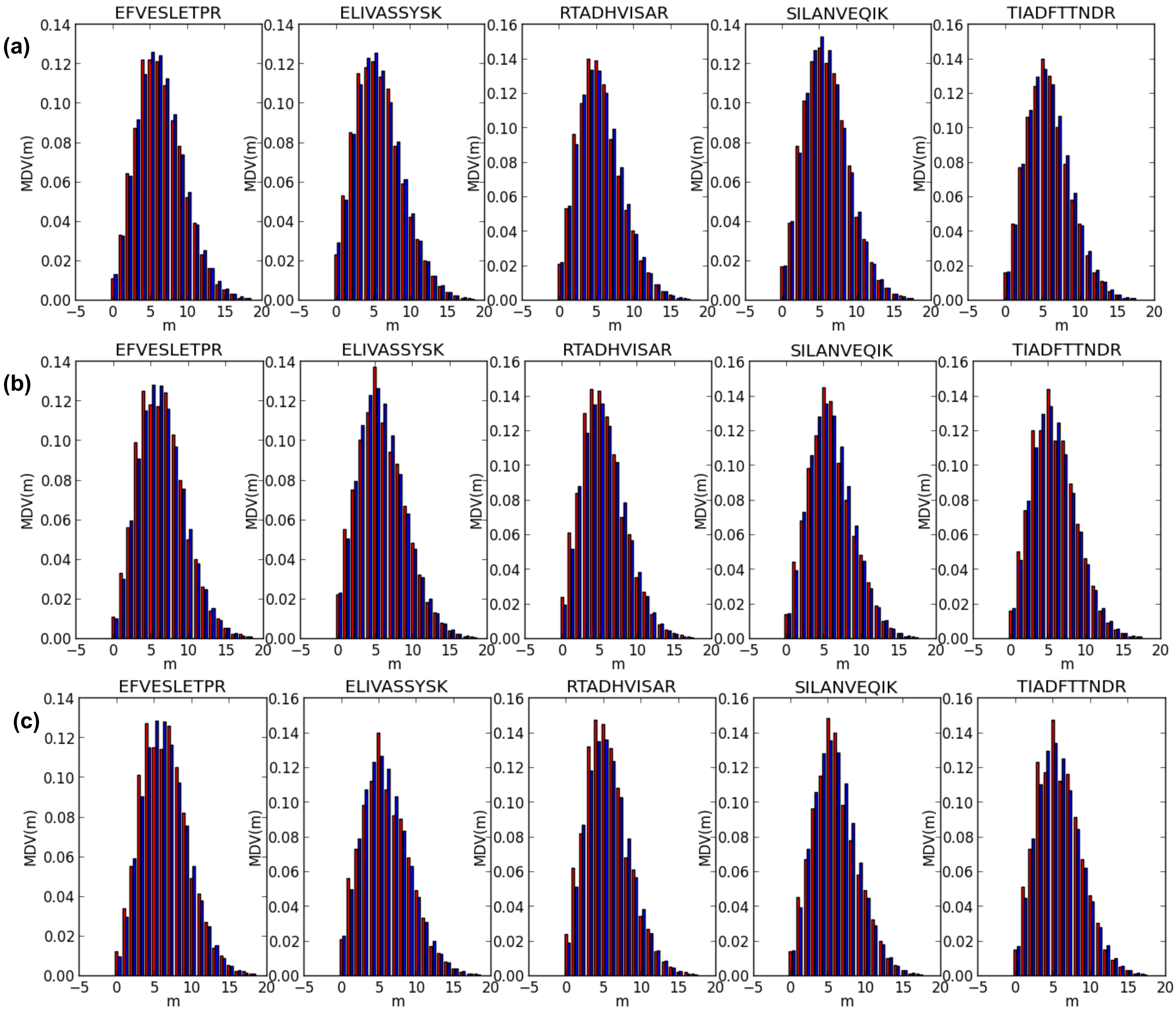

(a)
(b)
(c)
EFVESLETPR
ELIVASSYSK
RTADHVISAR
SILANVEQIK
TIADFTTNDR
MDV(m)
m

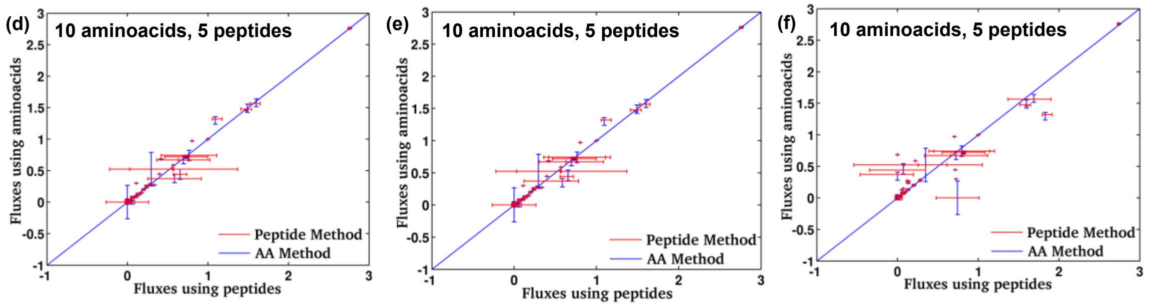

Figure S5. Peptide labeling data fit for the *E. coli* rf05 strain for 5 peptides and 10 amino acids. We have performed the same analysis as for figure S4 with following error rates (a) 0.05, (b) 0.08 and (c) 0.10, but using 5 peptides instead of 20. As can be observed the introduction noise has a much larger effect for this case than for the 20 peptides case.
