# Supplementary material for: A Peptide-Based Method for 13C Metabolic Flux Analysis in Microbial Communities
Source: PLoS Comput Biol. 2014 Sep 4;10(9):e1003827. doi: 10.1371/journal.pcbi.1003827 (PMC4154649; doi:10.1371/journal.pcbi.1003827)
Supplement: Figure S6 — Flux Information Content (FIC) for the amino acid and peptide-based 13C MFA for pgi knockout E. coli strain. The same trends as for the wild type can be observed, but in this case the number of required peptides is 15 instead of 20. (PDF) [file pcbi.1003827.s006.pdf]

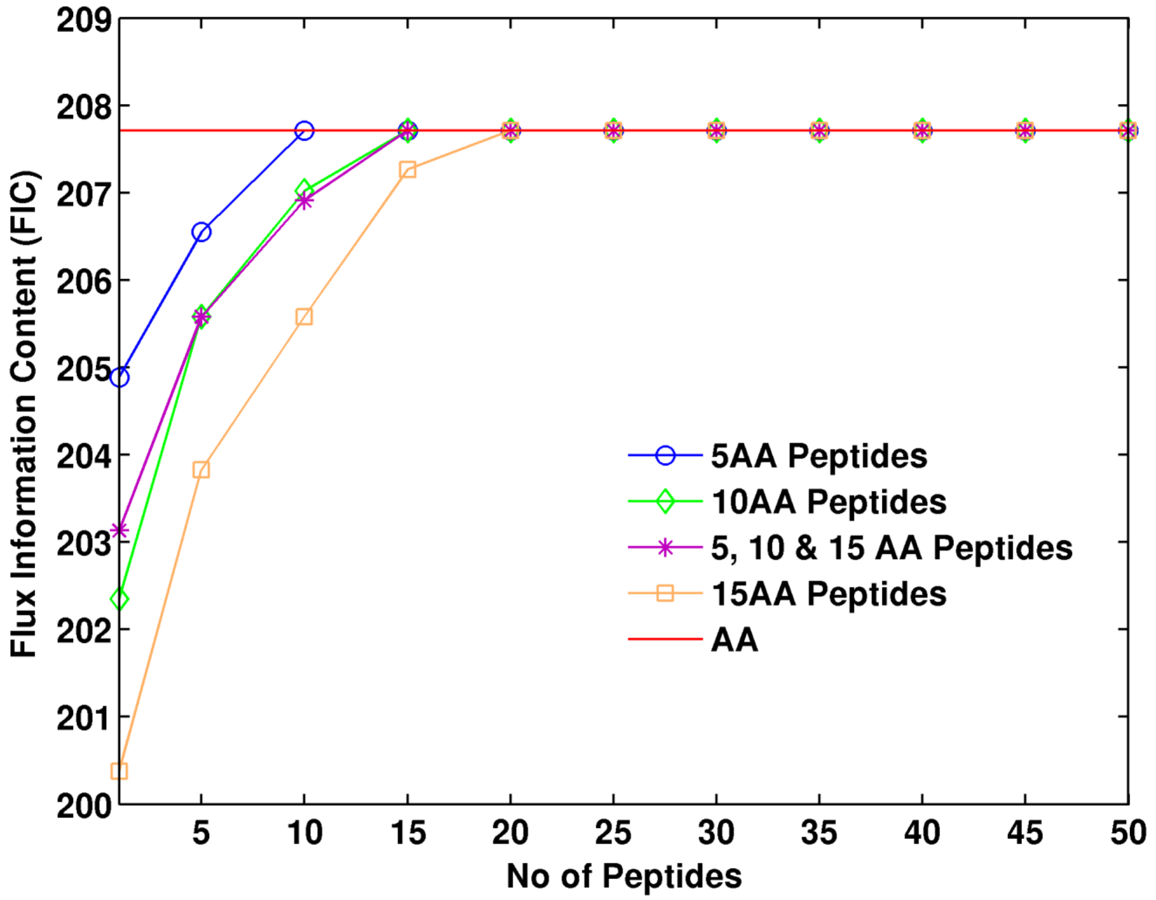

Figure S6. Flux Information Content (FIC) for the aminoacid and peptide-based ^{13}C MFA for pgi knockout *E. coli* strain. The same trends as for the wild type can be observed, but in this case the number of required peptides is 15 instead of 20.
