# Supplementary material for: A Peptide-Based Method for 13C Metabolic Flux Analysis in Microbial Communities
Source: PLoS Comput Biol. 2014 Sep 4;10(9):e1003827. doi: 10.1371/journal.pcbi.1003827 (PMC4154649; doi:10.1371/journal.pcbi.1003827)
Supplement: Figure S7 — Comparison between flux profiles obtained through the amino acid-based and the peptide-based 13C FMA for pgi knockout E. coli stain. Peptide based 13C FMA flux profile obtained for the best fit for peptide lengths of 5 amino acids. (PDF) [file pcbi.1003827.s007.pdf]

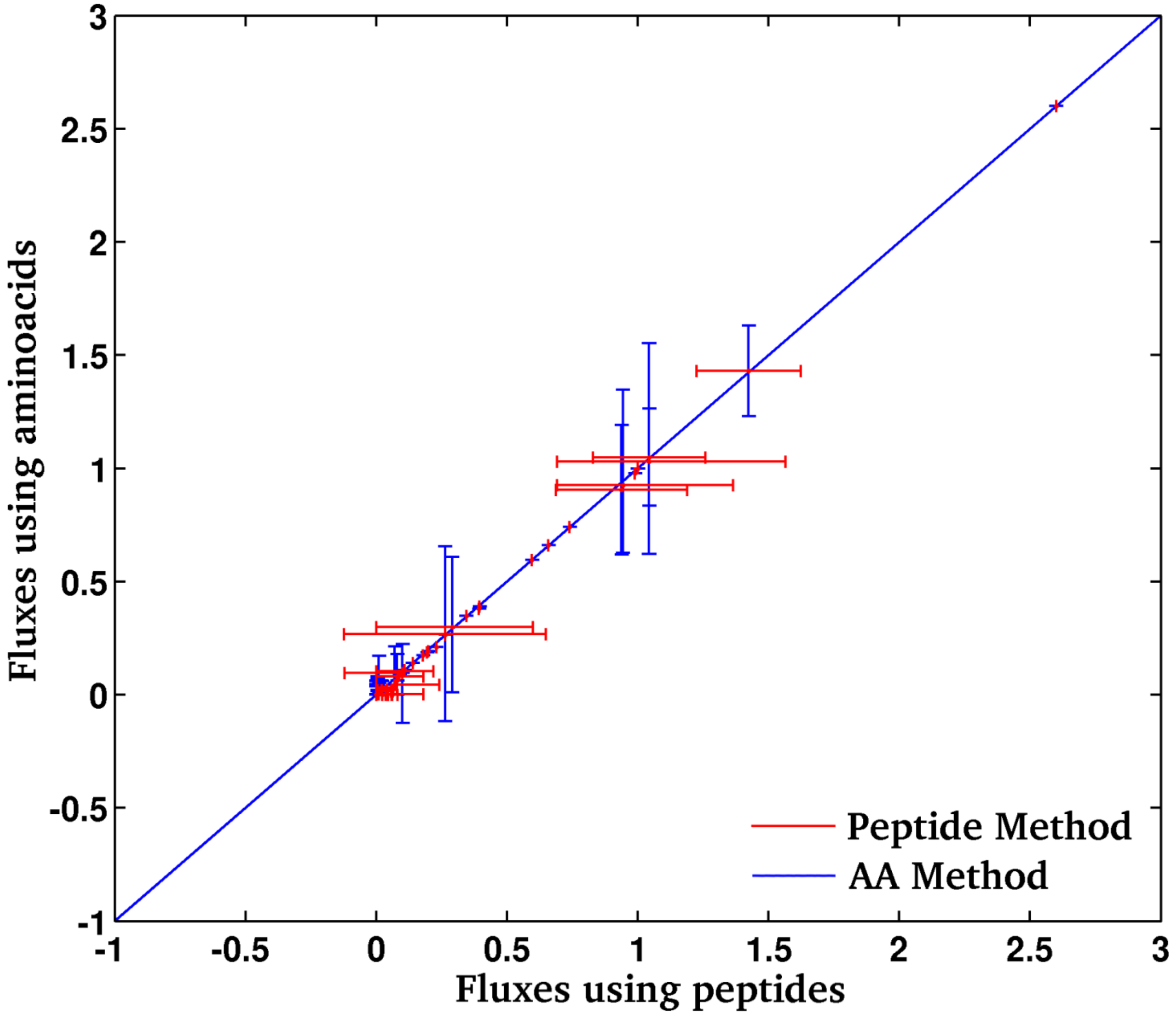

Figure S7. Comparison between flux profiles obtained through the amino acid-based and the peptide-based ^{13}C FMA for *pgi* knockout *E. coli* stain. Peptide based ^{13}C FMA flux profile obtained for the best fit for peptide lengths of 5 amino acids.
