# Supplementary material for: A Peptide-Based Method for 13C Metabolic Flux Analysis in Microbial Communities
Source: PLoS Comput Biol. 2014 Sep 4;10(9):e1003827. doi: 10.1371/journal.pcbi.1003827 (PMC4154649; doi:10.1371/journal.pcbi.1003827)
Supplement: Figure S8 — Peptide labeling labelling profile for peptide based 13C MFA obtained for D. vulgaris and M. maripaludis species in microbial community. Four 10 amino acids peptides sequences and MDV's were plotted for (a) DDFEPVNEVK, (b) NPEITDEENK, (c) GTALSGDDVR and (d) EGGTHLAGFK. (PDF) [file pcbi.1003827.s008.pdf]

(a)

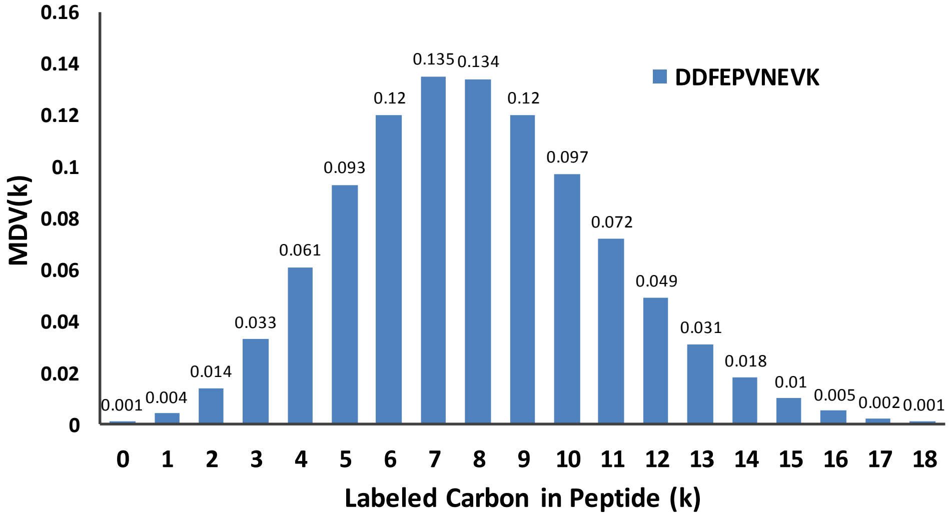
DDFEPVNEVK
MDV(k)
0 0.02 0.04 0.06 0.08 0.1 0.12 0.14 0.16
0.001 0.004 0.014 0.033 0.061 0.093 0.12 0.135 0.134 0.12 0.097 0.072 0.049 0.031 0.018 0.01 0.005 0.002 0.001
0 1 2 3 4 5 6 7 8 9 10 11 12 13 14 15 16 17 18
Labeled Carbon in Peptide (k)

(b)

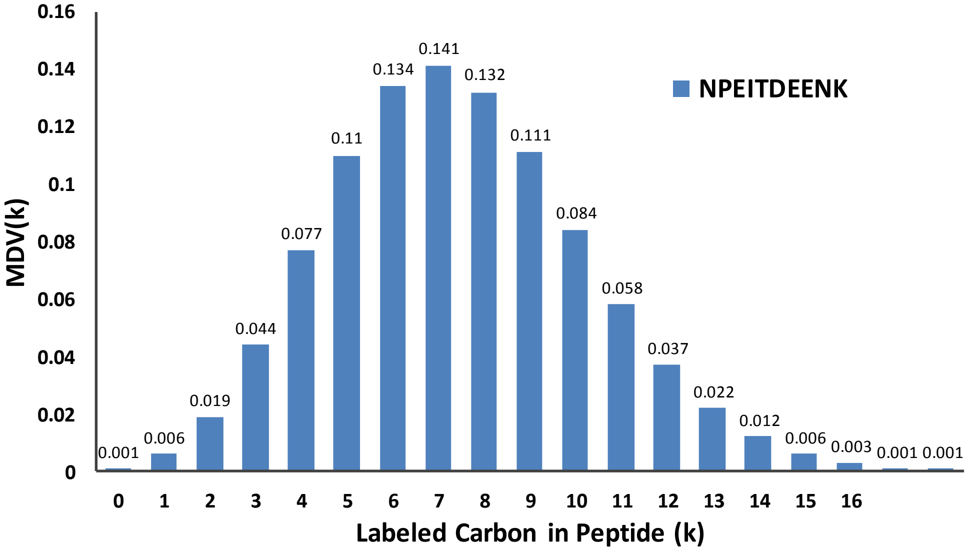
NPEITDEENK
MDV(k)
0 0.02 0.04 0.06 0.08 0.1 0.12 0.14 0.16
0.001 0.006 0.019 0.044 0.077 0.11 0.134 0.141 0.132 0.111 0.084 0.058 0.037 0.022 0.012 0.006 0.003 0.001 0.001
0 1 2 3 4 5 6 7 8 9 10 11 12 13 14 15 16
Labeled Carbon in Peptide (k)

(c)

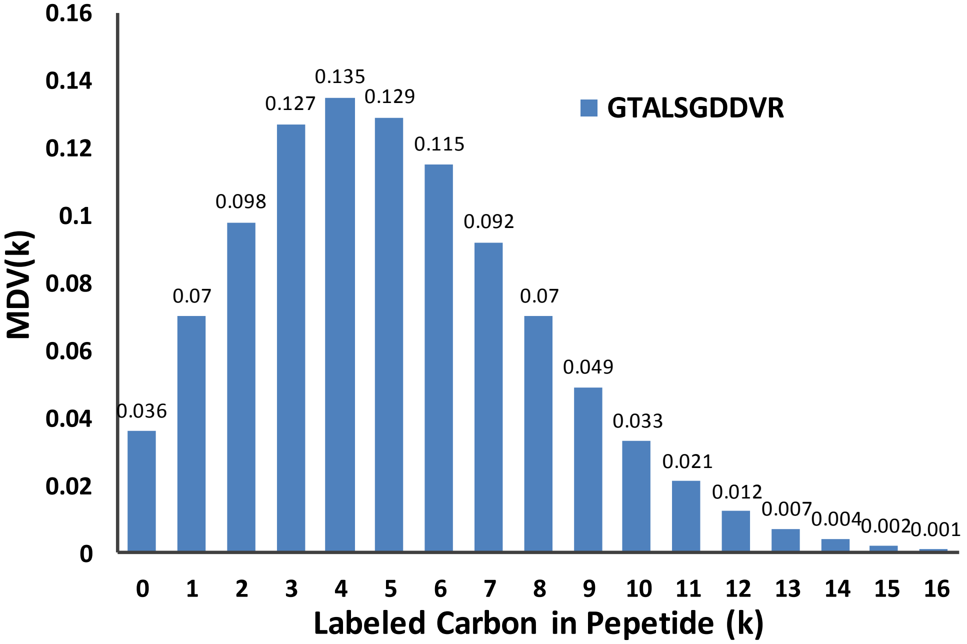

(d)

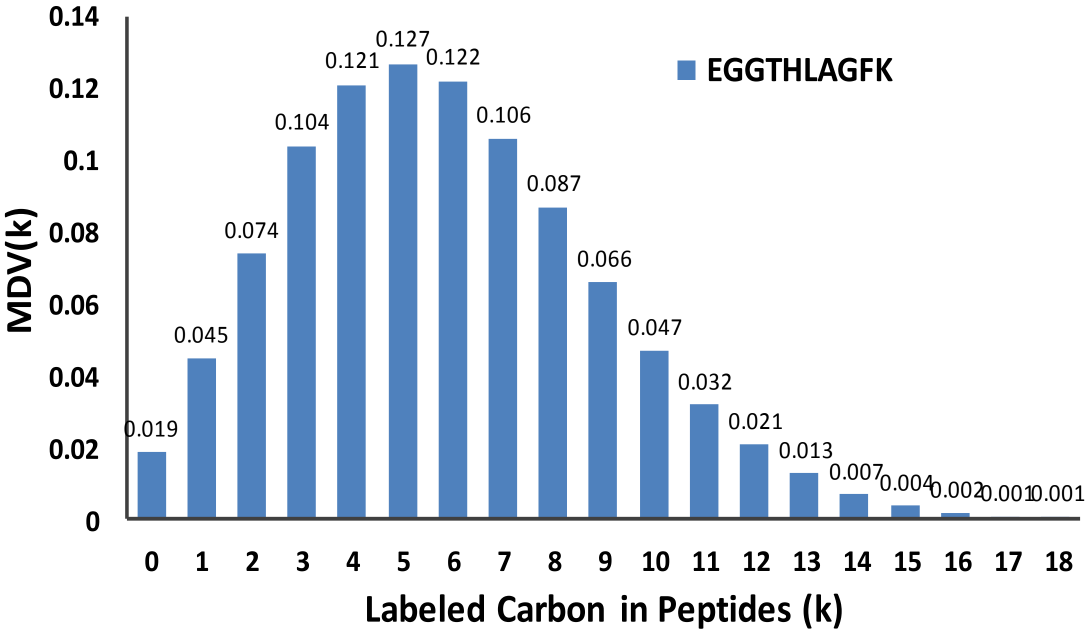

Figure S7. Peptide labeling labelling profile for peptide based 13C MFA obtained for *D. vulgaris* and *M. maripaludis* species in microbial community. Four 10 aminoacids peptides sequences and MDV's were plotted for (a) DDFEPVNEVK, (b) NPEITDEENK, (c) GTALSGDDVR and (d) EGGTHLAGFK.
