# Supplementary material for: A Peptide-Based Method for 13C Metabolic Flux Analysis in Microbial Communities
Source: PLoS Comput Biol. 2014 Sep 4;10(9):e1003827. doi: 10.1371/journal.pcbi.1003827 (PMC4154649; doi:10.1371/journal.pcbi.1003827)
Supplement: Table S1 — The following list comprises the reactions included in peptide and amino acid based 13C MFA methods, along with their corresponding carbon transitions. Carbon transitions indicate the fate of each carbon in the reaction. (PDF) [file pcbi.1003827.s009.pdf]

Table S1.

The following list comprises the reactions included in peptide and aminoacid based ^{13}C MFA methods, along with their corresponding carbon transitions. Carbon transitions indicate the fate of each carbon in the reaction.

	Reaction Name	*Reaction*	*Carbon transitions* obtained from Antoniewic *et al* [1]
	GLCpts:	glc-D[e] + pep → g6p + pyr	abcdef + ABC : abcdef + ABC
Glycolysis/gluconeogenesis			
	PGI:	g6p ↔ f6p	abcdef : abcdef
	PFK:	f6p → fdp	abcdef : abcdef
	FBA	fdp → g3p + dhap	CBAabc : abc + ABC
	TPI	dhap → g3p	abc : abc
	GAPD	g3p → 13dpg	abc : abc
	PGK	3pg → 13dpg	abc : abc
	PGM	2pg ↔ 3pg	abc : abc
	PYK	pep → pyr	abc : abc
	PDH	pyr → co2 + accoa	abc : a + bc
Pentose Phosphate Pathway			
	G6PDH2r	g6p → 6pgl	abcdef : abcdef
	PGL	6pgl → 6pgc	abcdef : abcdef
	GND	6pgc → co2 + ru5p-D	abcdef : a + bcdef
	RPE	ru5p-D ↔ xu5pD	abcde : abcde
	RPI	r5p ↔ ru5p-D	abcde : abcde
	TKT1	xu5pD + r5p ↔ g3p + s7p	ABCDE + abcde : CDE + ABabcde
	TALA	g3p + s7p ↔ f6p + e4p	ABC + abcdefg : abcABC + defg
	TKT2	xu5pD + e4p ↔ g3p + f6p	abABC + cdef : ABC + abcdef
	EDD	6pgc → 2ddg6p	abcdef : abcdef
	EDA	2ddg6p → pyr + g3p	abcdef : abc + def
Citric Acid Cycle/TCA			
	CS	oaa + accoa → cit	abcd + AB : dcbBAa
	ACONT	cit → icit	abcdef : abcdef
	ICDHy	icit → akg + co2	abcdef : abcde + f
	AKGDH	akg → succoa + co2	abcde : bcde + a
	SUCOAS	succ ↔ succoa	bcde : (bcde;edcb)
	SUCDi	succ → fum	abcd : abcd
	FUM	fum → mal-L	abcd : abcd
	MDH	mal-L ↔ oaa	abcd : abcd
Anaplerotic reactions			
	ENO	2pg ↔ pep	abc : abc
	PPC	pep + co2 → oaa	abc + d : abcd
	PPCK	oaa → pep + co2	abcd : abc + d
	ME1	mal-L → pyr + co2	abcd : abc + d
	ICL	icit → succ + glx	ABCDEF : FCDE + AB
	MALS	glx + accoa → mal-L	AB + CD : ABCD
Alanine and aspartate metabolism			
	ALATA_L	pyr → ala-L	abc : abc
	ASPTA	oaa → asp-L	abcd : abcd
	Asnsyn	aspL → asn-L	abcd : abcd
	Metsyn	aspL + mlthf → met-L	abcd + e : abcde
Glutamate metabolism			

GLUDy	glu-L ↔ akg	abcde : abcde
GLNS	glu-L → gln-L	abcde : abcde
Prosyn	gluL → pro-L	abcde : abcde
Argsyn	gluL + co2 → arg-L	abcde + f : abcdef
**Glycine and serine metabolism**		
PGCD	3pg → ser-L	abc : abc
GHMT2	ser-L → mlthf + gly	abc : c + ab
Cyssyn	serL → cysL	abc : abc
**Threonine and lysine metabolism**		
ASPK	asp-L → thrL	abcd : abcd
Lyssyn	aspL + pyr → lys-L + co2	abcd + efg : abcdgf + e
**Valine, leucine and isoleucine metabolism**		
ValSyn2	pyr + pyr → val-L + co2	abc + def : abcef + d
Ilesyn	thrL + pyr → ile-L + co2	abcd + efg : abfcdg + e
ACLS	pyr + accoa → pyrb	cde + ab : abde + c
LeuSyn	pyrb + pyr → leu-L + co2	abde + fgh : abdghe + f
**Tyrosine and phenylalanine metabolism**		
Presyn	pep + e4p → co2 +pre	def + ghij : d + efghij
PheSyn2	pre+ pep → phe-L	efghij + abc : abcefghij
Tyrsyn	pre + pep → tyr-L	efghij + abc : abcefghij
**Histidine and tryptophan metabolism**		
PRPPS	r5p + mlthf → his-L	abcde + f : edcbaf
Tipsyn	serL + r5p --> tip + g3p	abc + defgh : abced + fgh
Trpsyn	tip + pre --> trpL	abced + BCEFGH : abcedCEFGHB

Biomass fluxes:

G6Pbm	g6p → g6pbm	abcdef : abcdef
F6Pbm	f6p → f6pbm	abcdef : abcdef
R5Pbm	r5p → r5pbm	abcde : abcde
E4Pbm	e4p → e4pbm	abcd : abcd
G3Pbm	g3p → g3pbm	abc : abc
13dpgbm	13dpg → 13dpgbm	abc : abc
PEPbm	pep → pepbm	abc : abc
PYRbm	pyr → pyrbm	abc : abc
AcCoabm	accoa → accoabm	ab : ab
OAAbm	oaa → oaabm	abcd : abcd
AKGbm	akg → akgbm	abcde : abcde
CO2bm	co2 → co2bm	a : a

Fluxes out:

L_LACt2r	pyr → lacL	abc : abc
ADHer	accoa → etoh	ab : ab
ETOHt2r	etoh → etOHE	ab : ab
ACACCT	accoa → acE	ab : ab
