# Supplementary material for: A Peptide-Based Method for 13C Metabolic Flux Analysis in Microbial Communities
Source: PLoS Comput Biol. 2014 Sep 4;10(9):e1003827. doi: 10.1371/journal.pcbi.1003827 (PMC4154649; doi:10.1371/journal.pcbi.1003827)
Supplement: Table S2 — Flux values for amino acids and peptide method for 5 amino acids and 15 peptides. (PDF) [file pcbi.1003827.s010.pdf]

Table S2. Flux values for aminoacids and peptide method for 5 aminoacids and 15 peptides.

	Best Fit		Flux Range	
Rxns	AA	Peptides (5AA,1P)	AA	Peptides (5AA,1P)
13dpgbm	0.0900	0.1100	0.0200	0.0200
accoabm	0.2800	0.2800	0.0200	0.0200
akgbm	0.0700	0.0700	0.0200	0.0200
co2bm	2.7600	2.7600	0.0200	0.0200
e4pbm	0.0237	0.0381	0.0200	0.0200
f6pbm	0.0000	0.0000	0.0000	0.0000
FUM	0.6722	0.6722	0.1306	0.1306
g3pbm	0.0000	0.0000	0.0000	0.0000
g6pbm	0.0300	0.0300	0.0200	0.0200
oaabm	0.1300	0.1300	0.0200	0.0200
pepbm	0.0300	0.0300	0.0200	0.0200
pyrbm	0.2000	0.2000	0.0200	0.0200
r5pbm	0.0800	0.0600	0.0200	0.0200
ACACCT	0.0000	0.0000	0.0000	0.0000
ACLS	0.0001	0.0001	0.0599	0.0599
ACONT	0.7425	0.7425	0.1612	0.1612
ADHer	0.0000	0.0000	0.0000	0.0000
AKGDH	0.3726	0.3812	0.1306	0.1306
ALATA_L	0.0001	0.0001	0.0599	0.0599
ASPK	0.0001	0.0001	0.0596	0.0596
ASPTA	0.0004	0.0004	0.0596	0.0596
Argsyn	0.0001	0.0001	0.0597	0.0597
Asnsyn	0.0001	0.0001	0.0596	0.0596
CS	0.7425	0.7425	0.1612	0.1612
Cyssyn	0.0001	0.0001	0.0596	0.0596
EDA	0.0000	0.0000	0.0000	0.0000
EDD	0.0000	0.0000	0.0000	0.0000
ENO	1.4545	1.4545	0.0000	0.0000
ETOHt2r	0.0000	0.0000	0.0000	0.0000
FBA	0.7204	0.7304	0.0516	0.0516
G6PDH2r	0.5229	0.5229	0.0000	0.0000
GAPD	1.5656	1.5835	0.1251	0.1251
GHMT2	0.0209	0.0187	0.0596	0.0596
GLCpts	1.0000	1.0000	0.0000	0.0000
GLUDy	0.0003	0.0003	0.0597	0.0597
GND	0.5229	0.5229	0.0000	0.0000
Glnsyn	0.0001	0.0001	0.0597	0.0597
ICDHyr	0.4429	0.4515	0.1612	0.1612

	Best Fit		Flux Range	
Rxns	AA	Peptide (5AA,15P)	AA	Peptide (5AA,15P)
ICL	0.2996	0.2910	0.0000	0.0000
Ilesyn	0.0001	0.0001	0.0599	0.0599
L_LACt2r	0.0000	0.0000	0.0000	0.0000
LeuSyn	0.0001	0.0001	0.0599	0.0599
Lyssyn	0.0001	0.0001	0.0596	0.0596
MALS	0.2996	0.2910	0.0000	0.0000
MDH	0.9718	0.9633	0.0000	0.0000
ME1	0.0000	0.0000	0.5310	0.5310
Metsyn	0.0001	0.0001	0.0596	0.0596
PDH	1.3221	1.3136	0.1199	0.1199
PFK	0.7204	0.7304	0.0516	0.0516
PGCD	0.0211	0.0189	0.0596	0.0596
PGI	0.4471	0.4471	0.0000	0.0000
PGK	1.4756	1.4735	0.1326	0.1326
PGL	0.5229	0.5229	0.0000	0.0000
PGM	1.4545	1.4545	0.0000	0.0000
PPC	0.5862	0.5866	0.0000	0.0000
PPCK	0.6850	0.6768	0.0000	0.0000
PRPPS	0.0208	0.0186	0.0526	0.0526
PYK	0.5228	0.5143	0.5310	0.5310
PheSyn2	0.0001	0.0001	0.0467	0.0467
Presyn	0.0003	0.0003	0.0467	0.0467
Prosyn	0.0001	0.0001	0.0597	0.0597
RPE	0.2734	0.2833	0.0000	0.0000
RPI	0.2496	0.2396	0.0425	0.0425
SUCD1i	0.6722	0.6722	0.1306	0.1306
SUCOAS	0.3726	0.3812	0.0000	0.0000
TALA	0.1487	0.1608	0.0000	0.0000
THRAr	0.0001	0.0001	0.0596	0.0596
TKT1	0.1487	0.1608	0.0000	0.0000
TKT2	0.1247	0.1225	0.0000	0.0000
TPI	0.7204	0.7304	0.0238	0.0238
Tipsyn	0.0001	0.0001	0.0147	0.0147
Trpsyn	0.0001	0.0001	0.0147	0.0147
Tyrsyn	0.0001	0.0001	0.0443	0.0443
ValSyn1	0.0001	0.0001	0.0323	0.0323
ValSyn2	0.0001	0.0001	0.0323	0.0323
