# Supplementary material for: A Peptide-Based Method for 13C Metabolic Flux Analysis in Microbial Communities
Source: PLoS Comput Biol. 2014 Sep 4;10(9):e1003827. doi: 10.1371/journal.pcbi.1003827 (PMC4154649; doi:10.1371/journal.pcbi.1003827)
Supplement: Table S3 — List of Desulfovibrio vulgaris reactions included in peptide and amino acid based 13C MFA methods, along with their corresponding carbon transitions. (PDF) [file pcbi.1003827.s011.pdf]

Table S3. List of *Desulfovibrio vulgar*is reactions included in peptide and aminoacid based ^{13}C MFA methods, along with their corresponding carbon transitions.

	Reaction Name	*Reaction*	*Carbon transitions* obtained from Antoniewic *et al* [1]
	LACpts:	lac → pyr	abc : abc
Glycolysis/gluconeogenesis			
	PGI:	g6p ↔ f6p	abcdef : abcdef
	PFK:	f6p ↔ fdp	abcdef : abcdef
	FBA	fdp ↔ g3p + g3p	CBAabc : abc + ABC
	GPDH	g3p ↔ 3pg	abc : abc
	PGMTe	3pg ↔ pep	abc : abc
	PYK_f	pep → pyr	abc : abc
	PYK_b	pyr → pep	abc : abc
	PFOXDR	pyr ↔ co2 + accoa	abc : a + bc
	PYRCO2L	pyr + co2 → oaa	abc + d : abcd
Pentose Phosphate Pathway			
	G6PDH	g6p → co2 + ru5p-D	abcdef : a + bcdef
	RPE	ru5p-D ↔ xu5pD	abcde : abcde
	RPI	r5p ↔ ru5p-D	abcde : abcde
	TKT1	xu5pD + r5p ↔ g3p + s7p	ABCDE + abcde : CDE + ABabcde
	TALA	g3p + s7p ↔ f6p + e4p	ABC + abcdefg : abcABC + defg
	TKT2	xu5pD + e4p ↔ g3p + f6p	abABC + cdef : ABC + abcdef
Citric Acid Cycle/TCA			
	ACS	ac → accoa	ab : ab
	CS	oaa + accoa → cit	abcd + AB : dcbBAa
	CHL	cit ↔ icit	abcdef : abcdef
	ICDHyr	icit ↔ akg + co2	abcdef : abcde + f
	AKGS	akg ↔ succoa + co2	abcde : bcde + a
	SUCOAS	succ ↔ succoa	bcde : (bcde;edcb)
	SUCOXDR	succ ↔ fum	abcd : abcd
	FUM	fum ↔ mal-L	abcd : abcd
	MDH	mal-L ↔ oaa	abcd : abcd
Anaplerotic reactions			
	ME2	mal-L → pyr + co2	abcd : abc + d
	MALS	glx + accoa → mal-L	AB + CD : ABCD
Fluxes out			
	PFL	pyr ↔ accoa + for	abc : ab + c
	FDHfdx	for → co2	a : a
	EX_co2	co2 ↔ co2E	a : a
	GLYCL	gly ↔ co2 + mlthf	ab : a + b
	MTHFDH	mlthf --> for	a : a
	EX_for	for ↔ forE	a : a
	PTAr	accoa ↔ actp	ab : ab
	ACKr	actp ↔ ac	ab : ab
	EX_ac	ac ↔ acE	ab : ab
	GLYAK	g3p ↔ glyc3p	abc : abc
	GLYK	glyc3p → glyc	abc : abc
	EX_glyc	glyc ↔ glycE	abc : abc
Alanine and aspartate metabolism			
	ALATRA	pyr → ala-L	abc : abc
	ASPTA	oaa → asp-L	abcd : abcd

ASNS	aspL → asn-L	abcd : abcd
CYSMETT	homL + mlthf → metL	abcd + e : abcde

Glutamate metabolism

GLUD	akg → glu-L	abcde : abcde
GLNS	glu-L → gln-L	abcde : abcde
PROOX	gluL → pro-L	abcde : abcde
GLUACTR	gluL + co2 → arg-L	abcde + f : abcdef

Glycine and serine metabolism

PGDH	3pg → ser-L	abc : abc
GHMT2r	ser-L ↔ mlthf + gly	abc : c + ab
SERACT	serL → cysL	abc : abc

Threonine and lysine metabolism

HSDy	asa → homL	abcd : abcd
THRLS	homL → thrL	abcd : abcd
THRAr	thrL → gly + accoa	abcd : ab + cd
ASPSDH	aspL → asa	abcd : abcd
DHDPS	pyr + asa → ddp	efg + abcd : abcdgfe
DHDPRy	ddp → thdp	abcdefg : abcdefg
DAPDC	thdp → lysL + co2	abcdefg : abcdef + g

Valine, leucine and isoleucine metabolism

ValSyn	pyr → pyrb	abc : abc
ACLS	pyrb + pyr → alacS + co2	abc + def : abcef + d
KADTi	alacS → 3mob	abcde : abcde
VALTA	3mob → valL	abcde : abcde
MOBDH	accoa + 3mob → leuL + co2	ab + cdefg : abdfge + c
ACTTS	pyr + thrL → ileL + co2	efg + abcd : abfcdg + e

Tyrosine and phenylalanine metabolism

SKDH	pep + e4p ↔ skm	abc + ghij : abcghij
CHRS	pep + skm → chor	def + abcghij : abcdefghij
CHORM	chor → pphn	abcdefghij : abcdefghij
PPHNDH1	pphn → tyrL + co2	abcdefghij : abcefghij + d
PPHNDH2	pphn → pheL + co2	abcdefghij : abcefghij + d

Histidine and tryptophan metabolism

PRPPS	r5p + mlthf → his-L	abcde + f : edcbaf
Tipsyn	serL + r5p → tip + g3p	abc + defgh : abced + fgh
Trpsyn	tip + pre → trpL	abced + BCEFGH : abcedCEFGHB
AR5PT	r5p + chor → g3p + pyr + indole + co2	defgh + pqrijklmno : fgh + pqr + edklmnoj + i
TRPS2	serL + indole → trpL	abc + edklmnoj : abcedklmnoj
PRPPS	r5p + mlthf → hisL	abcde + f : edcbaf

Biomass fluxes:

G6Pbm	g6p → g6pbm	abcdef : abcdef
F6Pbm	f6p → f6pbm	abcdef : abcdef
R5Pbm	r5p → r5pbm	abcde : abcde
G3Pbm	g3p → g3pbm	abc : abc
PEPbm	pep → pepbm	abc : abc
AcCoabm	accoa → accoabm	ab : ab
CO2bm	co2 → co2bm	a : a
