# Supplementary material for: A Peptide-Based Method for 13C Metabolic Flux Analysis in Microbial Communities
Source: PLoS Comput Biol. 2014 Sep 4;10(9):e1003827. doi: 10.1371/journal.pcbi.1003827 (PMC4154649; doi:10.1371/journal.pcbi.1003827)
Supplement: Table S4 — List of Methanococcus maripaludis reactions included in peptide and amino acid based 13C MFA methods, along with their corresponding carbon transitions. (PDF) [file pcbi.1003827.s012.pdf]

Table S4. List of *Methanococcus maripaludis* reactions included in peptide and aminoacid based ^{13}C MFA methods, along with their corresponding carbon transitions.

	Reaction Name	*Reaction*	*Carbon transitions* obtained from Antoniewic *et al* [1]
	ACpts	ac → accoa	ab : ab
Glycolysis/gluconeogenesis			
	PGM	g6p ↔ g1p	abcdef : abcdef
	PGI	g6p ↔ f6p	abcdef : abcdef
	PFK	f6p ↔ fdp	abcdef : abcdef
	FBA	fdp ↔ g3p + g3p	CBAabc : abc + ABC
	GPDH	g3p ↔ 3pg	abc : abc
	PGMTe	3pg ↔ pep	abc : abc
	PYK_f	pep → pyr	abc : abc
	PYK_b	pyr → pep	abc : abc
	PFOXDR	pyr → co2 + accoa	abc : a + bc
	PYRCO2L	pyr + co2 → oaa	abc + d : abcd
Pentose Phosphate Pathway			
	RPI	xu5pD ↔ r5p	abcde : abcde
	TKT1	xu5pD + r5p ↔ g3p + s7p	ABCDE + abcde : CDE + ABabcde
	TALA	g3p + s7p ↔ f6p + e4p	ABC + abcdefg : abcABC + defg
	TKT2	xu5pD + e4p ↔ g3p + f6p	abABC + cdef : ABC + abcdef
Citric Acid Cycle/TCA			
	AKGS	akg ↔ succoa + co2	abcde : bcde + a
	SUCOAS	succ ↔ succoa	bcde : (bcde;edcb)
	SUCOXDR	succ ↔ fum	abcd : abcd
	FUM	fum ↔ mal-L	abcd : abcd
	MDH	mal-L ↔ oaa	abcd : abcd
Methane production			
	FDH	for --> co2	a : a
	FMFD	co2 --> formf	a : a
	CMOD	co2 --> co	a : a
	ACSCMD	co + ch3tmp --> accoa	a + b : ab
	FTHMPFT	formf --> formtp	a : a
	MTHMPC	formtp --> chtmp	a : a
	MTHMPD	chtmp --> ch2tmp	a : a
	MTHMPR	ch2tmp --> ch3tmp	a : a
	MTHMP	ch3tmp --> ch3CoM	a : a
	MCMR	ch3CoM --> ch4	a : a
Alanine and aspartate metabolism			
	ALATRA	pyr → ala-L	abc : abc
	ASPTA	oaa → asp-L	abcd : abcd
	ASNS	aspL → asn-L	abcd : abcd
	CYSMETT	homL + mlthf → metL	abcd + e : abcde
Glutamate metabolism			
	GLUD	akg → glu-L	abcde : abcde
	GLNS	glu-L → gln-L	abcde : abcde
	PROOX	gluL → pro-L	abcde : abcde
	GLUACTR	gluL + co2 → arg-L	abcde + f : abcdef
Glycine and serine metabolism			
	PGDH	3pg → ser-L	abc : abc
	GHMT2r	ser-L ↔ mlthf + gly	abc : c + ab
	SERACT	serL → cysL	abc : abc

Threonine and lysine metabolism

HSDy	asa → homL	abcd : abcd
THRLS	homL → thrL	abcd : abcd
ASPSDH	aspL → asa	abcd : abcd
DHDPS	pyr + asa → ddp	efg + abcd : abcdgfe
DHDPRy	ddp → thdp	abcdefg : abcdefg
DAPDC	thdp → lysL + co2	abcdefg : abcdef + g

Valine, leucine and isoleucine metabolism

ValSyn	pyr → pyrb	abc : abc
ACLS	pyrb + pyr → alacS + co2	abc + def : abcef + d
KADTi	alacS → 3mob	abcde : abcde
VALTA	3mob → valL	abcde : abcde
MOBDH	accoa + 3mob → leuL + co2	ab + cdefg : abdfge + c
ACTTS	pyr + thrL → ileL + co2	efg + abcd : abfcdg + e

Tyrosine and phenylalanine metabolism

SKDH	pep + e4p ↔ skm	abc + ghij : abcghij
CHRS	pep + skm → chor	def + abcghij : abcdefghij
CHORM	chor → pphn	abcdefghij : abcdefghij
PPHNDH1	pphn → tyrL + co2	abcdefghij : abcefghij + d
PPHNDH2	pphn → pheL + co2	abcdefghij : abcefghij + d

Histidine and tryptophan metabolism

PRPPS	r5p + mlthf → his-L	abcde + f : edcbaf
Tipsyn	serL + r5p → tip + g3p	abc + defgh : abced + fgh
Trpsyn	tip + pre → trpL	abced + BCEFGH : abcedCEFGHB
AR5PT	r5p + chor → g3p + pyr + indole + co2	defgh + pqrijklmno : fgh + pqr + edklmnoj + i
TRPS2	serL + indole → trpL	abc + edklmnoj : abcedklmnoj
PRPPS	r5p + mlthf → hisL	abcde + f : edcbaf

Biomass fluxes:

G3Pbm	g3p → g3pbm	abc : abc
AcCoabm	accoa → accoabm	ab : ab
Ilebm	ile-L → ilebm	abcdef : abcdef
Leubm	leu-L → leubm	abcdef : abcdef
Valbm	val-L → valbm	abcde : abcde
Tyrbm	tyr-L → tyrbm	abcdefghi : abcdefghi
Phebm	phe-L → phebm	abcdefghi : abcdefghi
