# Supplementary material for: A Peptide-Based Method for 13C Metabolic Flux Analysis in Microbial Communities
Source: PLoS Comput Biol. 2014 Sep 4;10(9):e1003827. doi: 10.1371/journal.pcbi.1003827 (PMC4154649; doi:10.1371/journal.pcbi.1003827)
Supplement: Table S5 — The Peptide sequences from trypsin digestion of D. vulgaris and M. maripaludis proteins. The peptide labeling profile of these sequences have been used to measure flux profiles for D. vulgaris and M. maripaludis strains in a community using the peptide-based method. (PDF) [file pcbi.1003827.s013.pdf]

Table S5. The Peptide sequences from trypsin digestion of *D. vulgaris* and *M.maripaludis* proteins. The peptide labeling profile of these sequences have been used to measure flux profiles for *D. vulgaris* and *M. maripaludis* strains in a community using the peptide-based method.

D. vulgaris **10AA**	***M.maripaludis*** **10AA**
GTALSGDDVR	DDFEPVNEVK
EGLTAVVSVK	NPEITDEENK
EGGTHLAGFK	FLDEIINIFK
SILENIEVLR	NMLTVSDAHK
AIMLELSDGR	ISIIASGGIR
LAEEEAAHAR	FGVNSEYLNK
IVHPLESLTR	EHPVWTPEVK
LEEALLQSEK	ACSFIESELK
IEVPGAAAIR	HIQEYTLDEK
GPMPPGAVMR	IFCDGGLYIK
AIEAPSSPEK	GIFEPENIEK
DSVVTCTVGR	GGSQPAIIEK
DYAPLLLLWK	VAILGAGCYR
KPLSSLPADK	DENWYVSVCR
QGDTLYSIAR	NVAVETVMTK
SAVNFTAGAR	FVIITQSGQK
LYEYENFGHK	SLEMLNNNEK
LDASGSSELR	IDENELNMIK
TALLDAGIVR	ILVTTNLLYR
AALEAVAADR	LQELGLEASR
